# Chitinase Expression in Listeria monocytogenes Is Influenced by *lmo0327*, Which Encodes an Internalin-Like Protein

**DOI:** 10.1128/AEM.01283-17

**Published:** 2017-10-31

**Authors:** Dafni Katerina Paspaliari, Vicky Gaedt Kastbjerg, Hanne Ingmer, Magdalena Popowska, Marianne Halberg Larsen

**Affiliations:** a Department of Veterinary and Animal Sciences, University of Copenhagen, Frederiksberg, Denmark; b National Food Institute, Technical University of Denmark, Kongens Lyngby, Denmark; c Department of Applied Microbiology, Institute of Microbiology, Faculty of Biology, University of Warsaw, Warsaw, Poland; University of Helsinki

**Keywords:** Listeria monocytogenes, chitin, chitinase, internalin, autolysin, murein hydrolase, Lmo0327, Lmo0325

## Abstract

The chitinolytic system of Listeria monocytogenes thus far comprises two chitinases, ChiA and ChiB, and a lytic polysaccharide monooxygenase, Lmo2467. The role of the system in the bacterium appears to be pleiotropic, as besides mediating the hydrolysis of chitin, the second most ubiquitous carbohydrate in nature, the chitinases have been deemed important for the colonization of unicellular molds, as well as mammalian hosts. To identify additional components of the chitinolytic system, we screened a transposon mutant library for mutants exhibiting impaired chitin hydrolysis. The screening yielded a mutant with a transposon insertion in a locus corresponding to *lmo0327* of the EGD-e strain. *lmo0327* encodes a large (1,349 amino acids [aa]) cell wall-associated protein that has been proposed to possess murein hydrolase activity. The single inactivation of *lmo0327*, as well as of *lmo0325* that codes for a putative transcriptional regulator functionally related to *lmo0327*, led to an almost complete abolishment of chitinolytic activity. The effect could be traced at the transcriptional level, as both *chiA* and *chiB* transcripts were dramatically decreased in the *lmo0327* mutant. In accordance with that, we could barely detect ChiA and ChiB in the culture supernatants of the mutant strain. Our results provide new information regarding the function of the *lmo0325-lmo0327* locus in L. monocytogenes and link it to the expression of chitinolytic activity.

**IMPORTANCE** Many bacteria from terrestrial and marine environments express chitinase activities enabling them to utilize chitin as the sole source of carbon and nitrogen. Interestingly, several bacterial chitinases may also be involved in host pathogenesis. For example, in the important foodborne pathogen Listeria monocytogenes, the chitinases ChiA and ChiB and the lytic polysaccharide monooxygenase Lmo2467 are implicated in chitin assimilation but also act as virulence factors during the infection of mammalian hosts. Therefore, it is important to identify their regulators and induction cues to understand how the different roles of the chitinolytic system are controlled and mediated. Here, we provide evidence for the importance of *lmo0327* and *lmo0325*, encoding a putative internalin/autolysin and a putative transcriptional activator, respectively, in the efficient expression of chitinase activity in L. monocytogenes and thereby provide new information regarding the function of the *lmo0325-lmo0327* locus.

## INTRODUCTION

Chitin is an insoluble aminopolysaccharide comprising repeated alternating *N*-acetylglucosamine (GlcNAc) units and is present in abundance in marine and soil environments (1). In these environments, chitin can constitute an important carbon and nitrogen nutrient source (2–4), and in accordance, a number of bacteria have been found to be chitinivorous (4, 5). Central to the scavenging and catabolism of chitin are chitinases and associated chitin-binding proteins/lytic polysaccharide monooxygenases (LPMOs), which coordinately allow the bacteria to degrade the chitin to assimilative chitooligosaccharides, including the *N*,*N*′-diacetylchitobiose [(GlcNAc)_2_] dimer and the GlcNAc monomer. Chitinases and LPMOs have recently been recognized as enzymes of high biotechnological potential (6–12). In addition, chitinases and LPMOs have, rather surprisingly, been implicated in the infectious processes of several chitinolytic pathogenic bacteria, where they act as virulence factors (reviewed in reference 13). The 2-fold significance of chitinolytic systems has led to increased efforts to describe and characterize these systems, their individual elements, and their mode of regulation.

In vibrios, dozens of proteins have been found to be involved in chitin sensing, chemotaxis, degradation, and assimilation (2, 14–18). In terms of degradation, these include extracellular enzymes, such as chitinases (16, 19) and the GlcNAc-binding protein GbpA (20), as well as periplasmic hydrolases that participate in the degradation of the oligosaccharides to the dimeric and monomeric subunits (21, 22). The components responsible for the recognition of chitin and subsequent induction of the chitinolytic system include a chitooligosaccharide-specific porin (23–25), encoded by *chiP*, as well as ChiS, a hybrid sensor kinase that is proposed to respond to chitooligosaccharides introduced into the periplasmic space through the action of ChiP and other porins (14).

ChiP, ChiS, and respective ChiR response regulator-like components have been identified in other Gram-negative chitinolytic bacteria as well, such as Serratia marcescens (26, 27), Escherichia coli (28), and Salmonella enterica serovar Typhimurium (29). Thus, it is possible that these sensing and regulatory elements are commonly used by chitinolytic Gram-negative bacteria in terms of chitin response.

In contrast, the periplasmic localization of some of these elements and the general role of the periplasmic space in the two-step (extracellular and periplasmic) degradation of chitin in Gram-negative bacteria imply that the chitin responses should be structurally different in their Gram-positive counterparts. Interestingly, a ChiSR two-component system involved in chitin response has also been described in the Gram-positive Streptomyces (30–32). To our knowledge, no analogues of ChiP have been described in Gram-positive bacteria so far. In general, little is known about the regulators of Gram-positive chitinolytic systems, although a number of Gram-positive chitinases and LPMOs have been identified and characterized (see for example, references 33–36).

Listeria monocytogenes is a Gram-positive foodborne pathogen that can cause listeriosis, a rare but potentially fatal disease. L. monocytogenes is chitinolytic (37), and its system is one of the best-characterized Gram-positive systems so far. Chitin hydrolysis relies on two chitinases, ChiA and ChiB, as well as an LPMO (Lmo2467) (37–39). These proteins have pleiotropic roles, contributing also to the colonization of eukaryotic unicellular and/or mammalian hosts (40–42). The expression of the two chitinases depends on the positive effects of the major L. monocytogenes regulators σ^B^ and PrfA, as well as the *agr* system (43, 44). At the same time, the small RNA LhrA exerts an inhibitory effect on the translation of *chiA* mRNA (45). Hfq and Lmo0106 appear to also play as yet undescribed roles in the regulation of chitinolytic activity (43, 45).

In this study, we employed a transposon screening system to identify novel components of the listerial chitinolytic system or regulators of chitinase activity. Our screen yielded *lmo0327* as a gene essential for the efficient expression of the chitinases. We additionally investigated the related putative transcriptional regulator Lmo0325 and found *lmo0325* to be also necessary for effective chitin hydrolysis.

## RESULTS

### Transposon screen of L. monocytogenes N53-1 for mutants with altered chitinase activity.

To identify regulators and proteins important for the chitinolytic activity of L. monocytogenes, we screened a *mariner*-based transposon library for mutants with an enhanced or impaired ability to hydrolyze chitin. The strain N53-1 was chosen for the screening, as we have found it to be a strain of relatively high chitinolytic potential, a trait that can facilitate the identification of mutants. The phenotypic evaluation was based on the size of the clearing zone produced after growth on erythromycin-selective LB-chitin agar plates.

**FIG 1:**
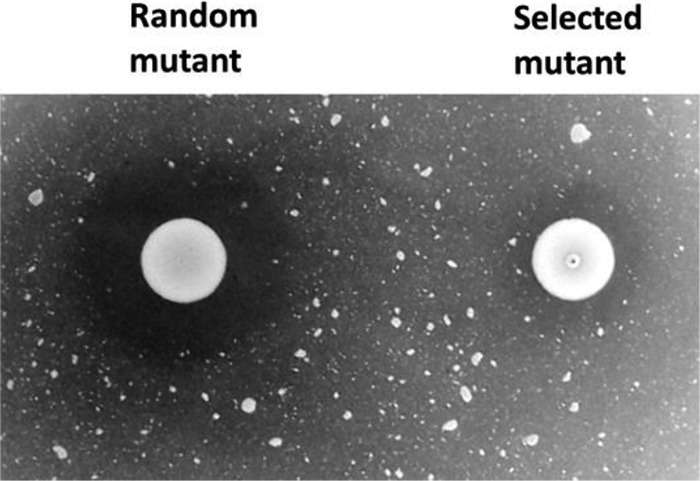
Identification of a transposon insertion mutant with impaired chitinolytic activity in strain N53-1. For screening purposes, the chitinolytic activity of random transposon mutants was evaluated by spotting overnight cultures of the mutants on LB-colloidal chitin agar plates containing erythromycin and incubating them for several days at 30°C. The overwhelming majority of the mutants exhibited strong chitinolytic activity, without great variance (left). In contrast, the chitinolytic activity of one of the mutants was greatly reduced (right).

A total of approximately 3,650 clones were screened. Among these, one mutant exhibited a dramatic decrease in chitinolytic activity (resembling Δ*chiA* mutants in the EGD strain) and was selected for further study (Fig. 1).

### Identification of the site of transposon insertion.

Given that the genome sequence available for strain N53-1 is not closed (GenBank accession number HE999705.1 [46]), we mapped the site of transposon insertion in relation to the genome of the well-annotated EGD-e strain instead (GenBank accession number NC_003210.1). The two strains are of the same serotype and their genomes have been found to be relatively similar (46).

**FIG 2:**
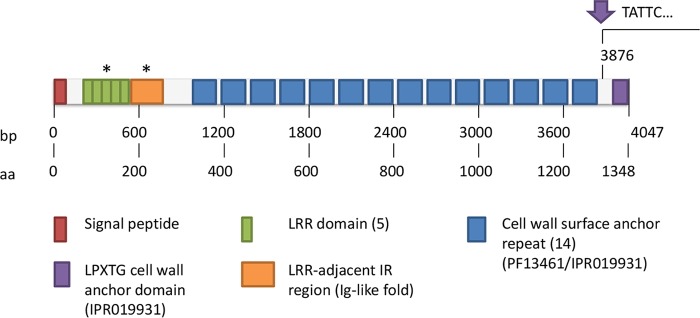
Structure of *lmo0327*/Lmo0327 with the arrow pointing to the position of the transposon insertion. The sequence of *lmo327* was obtained from ListiList (genolist.pasteur.fr/ListiList). Domain annotation was done based on the Pfam (http://pfam.xfam.org) and Uniprot (www.uniprot.org) databases, with the exception of the domains shown with asterisks, whose annotations were based on the structures reported by Popowska and Markiewicz (47) and Bierne and Cossart (53), respectively.

With respect to the genome of EGD-e, we found the transposon insertion to be between nucleotide 3875 and nucleotide 3876 of the 4,047-bp-long *lmo0327* (Fig. 2). *lmo0327* encodes a cell surface protein with proposed murein hydrolase activity (47). The protein has been classified as an internalin, as it carries the characteristic leucine-rich (LRR) repeats of the internalin family (48). Besides the repeats, the protein also harbors 14 cell wall surface repeats, as well as an LPXTG cell wall anchor domain. The position of the transposon insertion lies close to the C terminus, between the end of the cell wall surface anchor repeats and the LPXTG cell wall anchor domain. Although this insertion does not necessarily preclude the expression of the N-terminal part of the protein (47), it can be assumed to prevent the correct sorting of the protein and its anchoring to the cell wall. This should in turn be expected to abolish the activity of the protein.

The transposon insertion should not have caused any polar effects, as the coding region of *lmo0327* is flanked by terminators and a previous study has shown that insertional inactivation of the gene does not inhibit transcription of any of its upstream or downstream adjacent genes (47).

As the strain that was used for the transposon analysis was N53-1, we also tried to map the insertion site on the genome of this strain (genome accession number HE999705.1 [46]). Surprisingly, the corresponding locus differed from that of EGD-e. Namely, in the case of N53-1, the insertion appeared to be in an internalin J gene (NCBI locus tag BN419_0374), which in turn contained extensive deletions and a premature stop codon compared with the *lmo0327* locus in EGD-e. However, a careful examination revealed that these differences partly arose from the incomplete nature of the sequence provided for strain N53-1, leading to an erroneous annotation of the gene as internalin J, which in EGD-e is encoded by *lmo2821* (49). To confirm this, we sequenced the corresponding locus of N53-1. Compared to the EGD-e *lmo0327*, we found it to contain a single 414-bp (138-amino-acid [aa])-long in-frame deletion, confirmed also by PCR (results not shown). An alignment of the nucleotide and protein sequences between the two strains can be found in Fig. S2 and S3 in the supplemental material. The deletion corresponds to the area of the cell wall anchor repeats and likely does not influence the functionality of the protein.

### Inactivation of *lmo0327* results in reduced chitinolytic activity.

**FIG 3:**
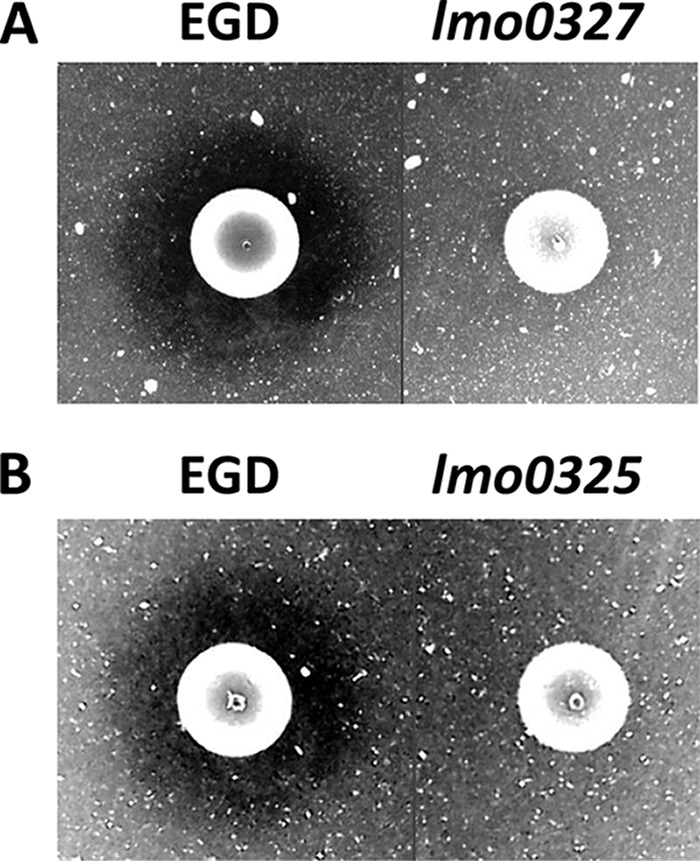
Chitinolytic activity of L. monocytogenes EGD on colloidal chitin agar plates depends on *lmo0327* (A) and *lmo0325* (B). The results are representative of at least three independent experiments. Small empty parts of the plates in panels A and B have been removed from the images, as indicated by the separating lines.

To confirm the phenotype, we performed a chitinolytic activity assay on the EGD strain and its corresponding *lmo0327* mutant, which carries an insertion that inactivates the Lmo0327 protein (47). Overnight cultures of the wild type and the mutant strain were spotted on colloidal chitin agar plates. After a 5-day incubation period at 30°C, the mutant strain displayed no visible clearing zone surrounding the colony, and slight hydrolysis could only be discerned upon colony removal (Fig. 3A). In contrast, the wild-type strain produced a clearly defined clearing zone (Fig. 3A). After prolonged incubation, a narrow clearing zone was also visible for the mutant (results not shown).

It should be noted here that all assays for the *lmo327* mutant were conducted in the absence of erythromycin to allow for a direct comparison with the wild-type EGD. As the assay was done under nonselective conditions, we compared the stabilities of the pAUL-A plasmid insertions in the absence of antibiotic selection under the conditions relevant for the assay. Although we recorded no loss of antibiotic resistance, the chitinolytic phenotype was slightly less pronounced than when the cells had been previously grown with erythromycin (results not shown). This suggests that the insertion was fairly stable but does not exclude that the actual differences may be even greater than those observed here.

### *lmo0325* mutant shows the same phenotype as the *lmo0327* insertion mutant.

In the EGD-e genome, *lmo0327* is preceded by *lmo0326* and *lmo0325*. Both of these genes are predicted to encode HTH-type Rgg transcriptional regulators (XRE family) that bear similarity to each other (47). Additionally, they have been suggested to both be essential for the function of Lmo0327, as their inactivation leads to the loss of autolytic activity attributed to Lmo0327 (47). In gel analyses of the surface proteins of EGD, the inactivation of *lmo0326* resulted in the disappearance of a protein band, which was hypothesized to correspond to Lmo0327 (47). This raises the possibility that *lmo0326* is a positive regulator of *lmo0327*. No similar analysis has been conducted for *lmo0325*. However, on the String database (string-db.org) for the prediction of protein interactions, it is suggested to interact with *lmo0327* based on the genomic neighborhood and cooccurrence across firmicute species.

These indications prompted us to investigate whether *lmo0325* might also be important for the chitinolytic phenotype. Indeed, upon comparison of an *lmo0325* mutant with the wild-type strain and the *lmo0327* mutant, we discovered that inactivation of *lmo0325* gave rise to a phenotype indistinguishable from that of the *lmo0327* mutant (Fig. 3B). This should not be due to polar effects on *lmo0327* resulting from the inactivation, as we found the transcription of both *lmo0326* and *lmo0327* to be unaffected by the insertion into *lmo0325* (data not shown).

### Amounts of chitinase transcripts are decreased in the *lmo0327* mutant.

**FIG 4:**
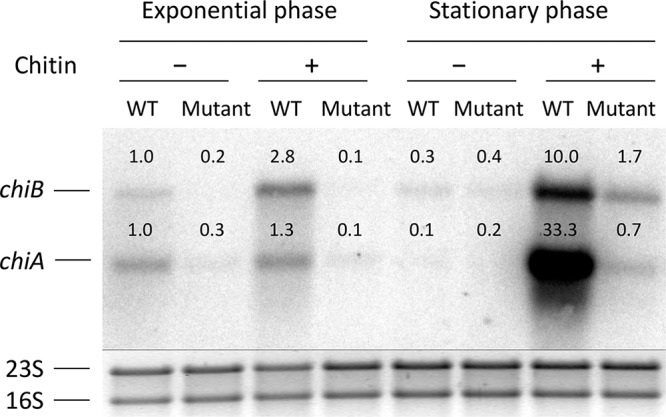
Northern blot analysis of *chiA* and *chiB* transcripts in the wild-type (WT) EGD and the *lmo0327* mutant. Samples were taken at mid-exponential and stationary phases in LB supplemented with 0.05% glucose and 1.7 g/liter colloidal chitin. The numbers above each band reflect the fold difference in relation to the respective transcript level of the first lane, i.e., the wild-type exponential-phase sample grown without chitin. The 16S and 23S loading controls, visualized on the gel prior to the transfer to the membrane, can be seen below each band. The controls have been positioned under the corresponding bands of the Northern blot hybridization to facilitate the interpretation of the results. The results are representative of three biological replicates harvested in the course of two independent experiments.

To investigate whether the reduced chitinolytic activity observed for the mutants was due to a decrease in chitinase production itself, we carried out a Northern blot analysis to compare the levels of chitinase transcripts between the wild-type EGD and the *lmo0327* mutant (Fig. 4).

From the Northern blot analysis, in general, it can be seen that the transcript levels of the chitinases in the mutant were lower than for the wild-type under all conditions that produced detectable wild-type transcripts and were very close to or below the detection limit of the assay. The differences were the greatest in stationary phase in the presence of chitin, namely, at the condition of maximum induction of the chitinases and, in particular, in the case of *chiA*.

Therefore, our results indicate that inactivation of *lmo0327* significantly diminishes the transcription of both chitinase genes.

### ChiA and ChiB production is decreased in the *lmo0327* mutant.

We additionally compared the extracellular levels of ChiA and ChiB in culture supernatants grown overnight in the presence of chitin. We employed two different types of analysis.

**FIG 5:**
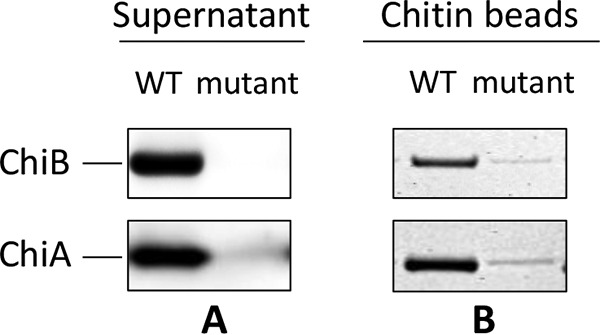
ChiA and ChiB levels in the supernatant depend on *lmo0327*. (A) Western blot analysis of overnight cultures of the wild type and the *lmo0327* insertion mutant. (B) SDS-PAGE analysis of ChiA and ChiB bound to chitin beads after incubation of the beads with overnight-culture supernatants of the wild type and the *lmo0327* insertion mutant. The results are representative of at least two independent experiments.

First, we compared by Western blot analysis the levels of overnight-secreted ChiA and ChiB between the wild type and the *lmo0327* mutant strain (Fig. 5A). For the amount of sample used for the analysis, we could detect no signal for either ChiA or ChiB in the culture supernatants of the mutant. In contrast, we detected strong signals in the wild-type supernatants. This confirms the results from the Northern blot and the phenotypic analysis, namely, that the production of both chitinases is greatly impaired upon inactivation of *lmo0327*.

It should be noted here, that the difference observed in the Western blot might be affected by the fact that a fraction of the chitinases remained bound to the chitin substrate after overnight incubation and was thereby not present in the supernatant visualized in the Western blot. However, we have previously shown that this limitation should not greatly influence the results, as first, there is still a significant amount of chitin remaining in the solution both in the mutant and in the wild-type cultures, and second, the amount of chitin-bound chitinases is relatively small compared to the amount of chitinases in the supernatant (44).

Still, the binding to chitin may be the reason why we could not detect any chitinase signal for the mutant strain at all, although small amounts of chitinases should still be produced, according to the phenotypic assay. To confirm the presence of ChiA and ChiB in the culture supernatants of the mutant, we incubated the supernatants from a larger culture volume together with chitin beads and analyzed the proteins bound to them by SDS-PAGE. This analysis revealed that both ChiA and ChiB are indeed produced at low levels by the *lmo0327* mutant, although this might also be related to the partial instability of the insertion (Fig. 5B). Additionally, despite not being a truly quantitative assay, the assay confirmed the difference in the levels of secreted chitinases between the wild type and the mutant that was observed in the Western blot.

## DISCUSSION

The biotechnological potential of bacterial chitinases (6–12), as well as their recognition as virulence factors of bacterial pathogens (13), has sparked an interest in deciphering the mode of function and regulation of bacterial chitinolytic systems. Although the structure and regulation of Gram-negative chitinolytic systems are being progressively elucidated, little is known about the regulation of the respective Gram-positive ones.

L. monocytogenes is one of the best-studied Gram-positive bacteria with respect to its chitinolytic system, and a number of regulators of chitinase activity have already been identified in the bacterium (43–45, 50). Despite these advances, the regulatory system of the listerial chitinases is expected to be more complex, including as of yet uncharacterized components. As an example, a conserved σ^54^ promoter box has been found in the promoter of *chiB*, pointing to σ^L^ being a *chiB* regulator (51).

To identify new regulatory components affecting chitinase activity, we carried out a *mariner*-based transposon screen on the strain N53-1, which exhibits strong chitinolytic activity. Our screening yielded one mutant severely impaired in chitin hydrolysis. The transposon insertion was mapped in a gene corresponding to the *lmo0327* locus in EGD-e, which is predicted to encode a protein of the internalin family with putative murein hydrolase activity (47, 48).

To confirm the implication of the gene in chitinase activity, we subsequently tested an EGD strain with insertional inactivation of *lmo0327*. Chitinolytic examination of this mutant on agar plates confirmed the phenotype exhibited by the transposon, as the mutant was only marginally positive for chitin hydrolysis. A similar phenotype was observed when the neighboring gene *lmo0325* was inactivated.

The fact that the inactivation of *lmo0327* affected chitinase expression in both the N53-1 and EGD strains suggests that this effect is not strain specific.

To investigate whether the inactivation affects a specific or both chitinases, we analyzed the *chiA* and *chiB* transcripts of the *lmo0327* mutant and the wild type grown with and without chitin. Under these conditions, chitin is necessary for induction of chitinase transcription (43). Our analysis revealed that, although chitin could still induce low-level transcription at stationary phase, both chitinase transcripts, and *chiA* in particular, were dramatically reduced in the mutant and near the detection limit of the assay. This suggests that *lmo327* affects chitinase expression at the transcriptional level. In agreement with this, neither ChiA nor ChiB was detected by Western blot of culture supernatants in the absence of *lmo0327*.

An incubation of a larger volume of *lmo0327* mutant culture supernatants with chitin beads ultimately revealed the presence of both chitinases in the supernatant, albeit at very low levels. This might indicate that chitinase production is not totally abolished in the mutant. Alternatively, it could be an artifact resulting from the instability of the pAUL-A insertion plasmid in a subpopulation of the cells.

When interpreting these results, the growth properties of the insertional mutant compared to those of the wild-type EGD-e should be taken into account. Namely, we recorded a deceleration in the growth rate of the mutant during the transition between late exponential and stationary phase (see Fig. S1 in the supplemental material). This difference might be related to the previously reported inhibition of cell separation exhibited by *lmo0327* mutant cells in exponential phase (47). In any case, the difference is not expected to greatly influence the results presented here.

The exact role of Lmo0327 in relation to the chitinolytic system, as well as its mechanism of action, is not easy to predict, as the function of the protein remains largely unknown. Its classification as an internalin does not provide much help in this case, as internalins have been proposed to be functionally diverse (48, 52). However, the presence of the LRR repeats might indicate a site for protein-protein interactions (53, 54). A role in virulence has been proposed for the protein, as it has been found to be important for intracellular replication in epithelial cells with an additional moderate role in adhesion/invasion (55). In agreement with that, the protein has been detected on the cell walls of intracellularly growing bacteria (56).

On the other hand, the protein had also previously been hypothesized not to contribute to virulence, as *lmo0327* is expressed at higher levels at temperatures relevant for environmental rather than host-associated growth (52, 57). *lmo0327* in general follows the temperature-expression patterns of σ^B^-dependent internalins (52). However, it has so far not been found to be σ^B^- or *prfA*-dependent (58–60), despite the presence of an imperfect *prfA* box in its promoter region (61). The absence of regulation by PrfA in rich medium should not exclude the possibility that *lmo0327* might be PrfA-dependent under specific conditions only, as is the case, for example, for the Listeria chitinases (43).

Interestingly, murein hydrolase activity has been suggested for Lmo0327, due to zymographic activity of extracts of cells expressing a gene fragment composed of *lmo0325*, *lmo0326*, and the N-terminal part of *lmo0327* (47). All three of these genes, but not *lmo0324* that was additionally tested, are important for this activity. However, as the other two genes are transcriptional regulators, the activity has been attributed to Lmo0327. In support of this, a band of autolytic activity corresponding to the size of Lmo0327 is detected in cell wall extracts of the wild-type EGD but is absent in mutant cells with inactivated *lmo0327* (47). In addition, mutants lacking *lmo0327* and *lmo0326* (its putative positive regulator) show an impaired ability to separate in exponential phase, as well as a lower rate of murein turnover (47). All this supports that Lmo0327 is an autolysin. However, more experimental data are needed to provide concrete proof, especially in view of the fact that Lmo0327 does not contain any identified domain associated with peptidoglycan hydrolysis and does not exhibit similarity with autolysins on basic homology searches.

The autolytic activity suggested for Lmo0327 raises the possibility of direct involvement of Lmo0327 in chitin hydrolysis, given the structural similarity between chitin and murein. The identification of the domain responsible for murein hydrolysis should aid in the examination of this possibility. However, it is more likely that chitin hydrolysis is affected solely by the effect exerted by *lmo0327* on chitinase transcription and thus on expression. It would be tempting to imagine that based on its surface location, the presence of the LRR repeats, and the transcriptional downstream effects, Lmo0327 could be a sensor, playing roles similar to those of ChiS and inducing chitinase transcription. However, this scenario is rendered unlikely by the absence of an established sensor-related domain in Lmo0327. It could also be that the effect of *lmo0327* on chitinase production is indirect. More experimental data are necessary to formulate further hypotheses.

In conclusion, we provide here evidence that *lmo0327* and *lmo0325*, encoding a putative internalin/autolysin and a putative transcriptional activator, respectively, are necessary for the efficient expression of chitinase activity in L. monocytogenes. The identification of yet another completely novel element affecting the chitinolytic system suggests that the system is even more complicated than previously anticipated and probably entails a great deal of interplay between individual regulatory factors to ensure the proper and timely expression of the chitinases.

## MATERIALS AND METHODS

### Bacterial strains and standard growth conditions.

The L. monocytogenes EGD wild type and its mutant derivative with a pAUL-A (Erm^r^) insertion in *lmo0327* were provided by Magdalena Popowska (47). The N53-1 strain was obtained from Lone Gram (62).

A mutant with a pAUL-A insertion in *lmo0325* was constructed in the same way as the *lmo0327* mutant, using the primer set 5′-TTGGATTGATTTCGTCGAGCTATC-3′ and 5′-CCACTTCGCCTTCTTTCATCCA-3′ (47). The nonpolar effects of the insertion on *lmo0326* and *lmo0327* were confirmed by reverse transcriptase PCR (RT-PCR), as described previously (47).

Bacteria were routinely grown aerobically in brain heart infusion (BHI) broth (Oxoid) at 37°C, unless stated otherwise. To draw growth curves of the wild-type and mutant strains, the strains were first grown aerobically in Lennox broth (LB) (BD Difco) at 30°C and 200 rpm overnight. The next day, the cultures were diluted in LB supplemented with 0.05% glucose to an optical density at 600 nm (OD_600_) of 0.05, and thereafter grown at 30°C and 190 rpm. Growth was monitored by periodically measuring the OD_600_ in an Eppendorf BioPhotometer Plus, as well as by determining the number of CFU/ml by spreading serial dilutions in 0.9% saline on BHI agar plates.

### Colloidal chitin preparation.

Colloidal chitin was prepared from chitin from shrimp shells (no. C9213; Sigma) as described previously (44).

### Construction of a *mariner*-based transposon library.

The *mariner*-based transposon delivery plasmid pMC38 was used for the generation of a random mutant bank of L. monocytogenes N53-1, as described by Cao et al. (63). Competent L. monocytogenes N53-1 cells were prepared and electroporation was performed as described by Kastbjerg et al. (64). Cells were spread on BHI agar with erythromycin (5 μg/ml). The L. monocytogenes N53-1 harboring pMC38 was grown overnight in BHI broth with erythromycin (5 μg/ml) and kanamycin (10 μg/ml) at 30°C with shaking. The cultures were diluted 1:200 in BHI broth with erythromycin (5 μg/ml) and grown for 1 h at 30°C with shaking, and then shifted to 40°C to force transposon integration for approximately 6 h until the OD_600_ was between 0.3 and 0.5. To select for chromosomal integration of the transposon, the cultures were grown at 40°C on BHI agar plates supplemented with erythromycin (5 μg/ml) for 3 to 4 days.

### Southern hybridization.

To evaluate the randomness of transposition, we arbitrarily picked 18 erythromycin-resistant colonies from our library. Genomic DNA was purified from the 18 isolates and from wild-type L. monocytogenes N53-1 using the Fast DNA kit (no. 116540-400; MP Biomedicals) as described by Holch et al. (46). DNA was precipitated with ethanol, and 4 μg DNA was digested with 20 U HindIII (Promega) for 3 h at 37°C. A 400-bp-long fragment of *ermC* of the transposon was amplified as a DNA probe, as described by Cao et al. (63). Labeling of the fragment and DNA hybridization were performed according to the protocol supplied with the biotin DecaLabel DNA labeling kit (no. K0652; Fermentas) and the biotin chromogenic detection kit (no. K0662; Fermentas).

### Transposon screening.

Cells from the frozen stock of the N53-1 *mariner*-based transposon library were either streaked on BHI agar plates containing 5 μg/ml erythromycin or diluted in 0.9% saline and then plated on the plates. After overnight growth at 37°C, individual clones were picked with toothpicks and spotted on assay plates (LB agar plates containing 6 mg/ml acid-hydrolyzed colloidal chitin and 5 μg/ml erythromycin). The clones were compared based on the size of the clearing zone produced after approximately 5 days of incubation at 30°C. Clones that seemed to exhibit an altered pattern of hydrolysis compared with that of the average were selected for further testing. This consisted of growing them overnight at 30°C under aerobic conditions (200 rpm) in BHI supplemented with 5 μg/ml erythromycin and spotting 10 μl of overnight culture on assay plates. The size of the clearing zone was recorded after 4 to 6 days of growth at 30°C. Clones that produced zones larger or narrower than the average were kept as frozen stocks containing 15% glycerol.

### Identification of the site of transposon insertion.

To identify the site of transposon insertion, we used the method described by Cao et al. (63), consisting of two rounds of arbitrary PCR aimed at amplifying the DNA sequences flanking the transposon and sequencing of the resulting products (Macrogen, Europe). However, in contrast to that by Cao et al. (63), Dream*Taq* Green DNA polymerase (Thermo Scientific) was used for the amplifications.

### Sequencing of the *lmo0327* locus of strain N53-1.

Chromosomal DNA from strain N53-1 was extracted with the aid of the DNeasy blood and tissue kit (Qiagen), according to the manufacturer's instructions. Sequencing was carried out by Macrogen, Europe.

### Determination of chitinolytic activity.

The wild type and the *lmo0325* and *lmo0327* mutant strains were grown overnight in BHI broth, which in the case of the mutants, had been supplemented with 5 μg/μl erythromycin. The next day, they were spotted on LB-chitin agar plates without erythromycin. After a 5-day incubation period at 30°C, the sizes of the produced clearing zones were compared between the wild type and the mutant.

### Sample collection for total RNA extraction.

To collect samples for RNA extraction, the cells were grown aerobically in LB at 30°C and 200 rpm overnight. The next day, the cultures were diluted in LB supplemented with 0.05% glucose to an OD_600_ of 0.05, and then grown at 30°C and 190 rpm. After 1 h of growth, half of the culture from each strain was induced by the addition of colloidal chitin to a final concentration of 1.7 g/liter. The cells were then grown until late exponential phase and samples for RNA were collected at an OD_600_ 0.7 for the wild-type EGD and 0.65 for the *lmo0327* mutant from both induced and uninduced cells. Stationary-phase samples were collected approximately 1 h after growth had been arrested, as indicated by OD_600_ measurements (approximately after 6 h of growth for the wild type and after 9 to 10 h of growth for the mutant) (see Fig. S1 in the supplemental material).

### Total RNA extraction and Northern blot analysis.

Total RNA extraction and Northern blot analysis were carried out as described previously for the Northern blot analysis of *agrA* transcripts (44). The radioactive probes were generated by PCR amplification using the primers described by Larsen et al. (43) Differences in the amounts of transcripts were considered to be significant only if they exceeded 2-fold.

### Protein precipitation of bacterial supernatants.

To collect samples of chitin-induced cells, the cells were initially grown aerobically in LB at 30°C and 200 rpm. Following overnight incubation, the cultures were diluted in LB supplemented with 0.05% glucose to an OD_600_ of 0.05 and then grown at 30°C and 190 rpm. After approximately 4 h of growth, 10 ml of the culture was induced by transferring to flasks containing 0.1 g of colloidal chitin. The induced and uninduced cells were left to grow overnight at 30°C and 190 rpm.

The next day, the OD_600_ of the uninduced cells was recorded. Nine milliliters of induced cells was spun down at 6,000 rpm at 0°C for 7 min. The supernatants were transferred to new tubes and the proteins were precipitated with trichloroacetic acid (Sigma-Aldrich) and normalized as described by Paspaliari et al. (44).

### Western blot analysis.

For Western blot analysis, 2 μl from samples was separated in a 4 to 12% NuPAGE Bis-Tris gel (Invitrogen) running with MOPS (morpholinepropanesulfonic acid) buffer. Thereafter the separated samples were transferred to a polyvinylidene difluoride (PVDF) membrane with the iBlot dry blotting system (Invitrogen). Western blot analysis was carried out with the aid of the WesternBreeze chemiluminescent kit (anti-rabbit; Invitrogen), according to the manufacturer's instructions. The anti-ChiA and anti-ChiB antibodies used for the analysis were obtained from Paspaliari et al. (44).

### SDS-PAGE analysis of extracellular proteins bound to chitin beads.

For the Western blot, the cells were initially grown aerobically in LB at 30°C and 200 rpm overnight. The next day, the cultures were diluted in 23 ml of LB supplemented with 0.05% glucose to an OD_600_ of 0.05 and thereafter grown at 30°C and 190 rpm. After approximately 1 h of growth, the cells were induced by the addition of colloidal chitin (10 g/liter) and left to grow overnight. The overnight-grown cells were separated from the supernatant by centrifugation. The supernatant was sterile-filtered using a 0.2-μm-pore-size sterile filter (Millipore) and incubated overnight with 75 μl of chitin magnetic beads (New England BioLabs) at 30°C and 200 rpm to allow binding of the chitinases to the beads. The next day, the beads were separated with the aid of a magnet and washed twice in 1 ml 50 mM Tris, pH 8.0. The washed beads were then resuspended in 20 μl of SDS-PAGE loading buffer (Invitrogen) and boiled for 10 min at 99°C to release the proteins. The proteins were separated on a 10% NuPAGE Bis-Tris gel (Invitrogen) and stained with SYPRO Ruby (Invitrogen), according to the manufacturer's instructions.

### Bioinformatic analyses.

All bioinformatic analyses were carried out using the CLC Main Workbench software (CLC Bio).

### Accession number(s).

The sequence of the gene in which the transposon had been inserted in the N53-1 strain was deposited in GenBank under the accession number MF314113.

## Supplementary Material

Supplemental material

## References

[1] Gooday GW 1990 The ecology of chitin degradation, p 387–430. *In* Marshall KC (ed), Advances in microbial ecology, vol 11 Plenum Press, New York, NY.

[2] Keyhani NO, Roseman S 1999 Physiological aspects of chitin catabolism in marine bacteria. Biochim Biophys Acta 1473:108–122. doi:10.1016/S0304-4165(99)00172-5. 10580132

[3] Aluwihare LI, Repeta DJ, Pantoja S, Johnson CG 2005 Two chemically distinct pools of organic nitrogen accumulate in the ocean. Science 308:1007–1010. doi:10.1126/science.1108925. 15890880

[4] Beier S, Bertilsson S 2013 Bacterial chitin degradation-mechanisms and ecophysiological strategies. Front Microbiol 4:149. doi:10.3389/fmicb.2013.00149. 23785358 PMC3682446

[5] Gooday GW 1990 Physiology of microbial degradation of chitin and chitosan. Biodegradation 1:177–190. doi:10.1007/BF00058835.

[6] Hoell IA, Vaaje-Kolstad G, Eijsink VGH 2010 Structure and function of enzymes acting on chitin and chitosan. Biotechnol Genet Eng Rev 27:331–366. doi:10.1080/02648725.2010.10648156. 21415904

[7] Dahiya N, Tewari R, Hoondal GS 2006 Biotechnological aspects of chitinolytic enzymes: a review. Appl Microbiol Biotechnol 71:773–782. doi:10.1007/s00253-005-0183-7. 16249876

[8] Bhattacharya D, Nagpure A, Gupta RK 2007 Bacterial chitinases: properties and potential. Crit Rev Biotechnol 27:21–28. doi:10.1080/07388550601168223. 17364687

[9] Eijsink VGH, Vaaje-Kolstad G, Vårum KM, Horn SJ 2008 Towards new enzymes for biofuels: lessons from chitinase research. Trends Biotechnol 26:228–235. doi:10.1016/j.tibtech.2008.02.004. 18367275

[10] Nagpure A, Choudhary B, Gupta RK 2013 Chitinases: in agriculture and human healthcare. Crit Rev Biotechnol 34:215–232. doi:10.3109/07388551.2013.790874. 23859124

[11] Aam BB, Heggset EB, Norberg AL, Sørlie M, Vårum KM, Eijsink VGH 2010 Production of chitooligosaccharides and their potential applications in medicine. Mar Drugs 8:1482–1517. doi:10.3390/md8051482. 20559485 PMC2885077

[12] Vaaje-Kolstad G, Westereng B, Horn SJ, Liu Z, Zhai H, Sørlie M, Eijsink VGH 2010 An oxidative enzyme boosting the enzymatic conversion of recalcitrant polysaccharides. Science 330:219–222. doi:10.1126/science.1192231. 20929773

[13] Frederiksen RF, Paspaliari DK, Larsen T, Storgaard BG, Larsen MH, Ingmer H, Palcic MM, Leisner JJ 2013 Bacterial chitinases and chitin-binding proteins as virulence factors. Microbiology 159:833–847. doi:10.1099/mic.0.051839-0. 23519157

[14] Li X, Roseman S 2004 The chitinolytic cascade in vibrios is regulated by chitin oligosaccharides and a two-component chitin catabolic sensor/kinase. Proc Natl Acad Sci U S A 101:627–631. doi:10.1073/pnas.0307645100. 14699052 PMC327198

[15] Hunt DE, Gevers D, Vahora NM, Polz MF 2008 Conservation of the chitin utilization pathway in the *Vibrionaceae*. Appl Environ Microbiol 74:44–51. doi:10.1128/AEM.01412-07. 17933912 PMC2223224

[16] Meibom KL, Li XB, Nielsen AT, Wu C, Roseman S, Schoolnik GK 2004 The *Vibrio cholerae* chitin utilization program. Proc Natl Acad Sci U S A 101:2524–2529. doi:10.1073/pnas.0308707101. 14983042 PMC356983

[17] Li X, Wang L-X, Wang X, Roseman S 2007 The chitin catabolic cascade in the marine bacterium *Vibrio cholerae*: characterization of a unique chitin oligosaccharide deacetylase. Glycobiology 17:1377–1387. doi:10.1093/glycob/cwm096. 17884842

[18] Pruzzo C, Vezzulli L, Colwell RR 2008 Global impact of *Vibrio cholerae* interactions with chitin. Environ Microbiol 10:1400–1410. doi:10.1111/j.1462-2920.2007.01559.x. 18312392

[19] Connell TD, Metzger DJ, Lynch J, Folster JP 1998 Endochitinase is transported to the extracellular milieu by the *eps*-encoded general secretory pathway of *Vibrio cholerae*. J Bacteriol 180:5591–5600. 9791107 10.1128/jb.180.21.5591-5600.1998 PMC107616

[20] Kirn TJ, Jude BA, Taylor RK 2005 A colonization factor links *Vibrio cholerae* environmental survival and human infection. Nature 438:863–866. doi:10.1038/nature04249. 16341015

[21] Keyhani NO, Roseman S 1996 The chitin catabolic cascade in the marine bacterium *Vibrio furnissii*. Molecular cloning, isolation, and characterization of a periplasmic beta-*N*-acetylglucosaminidase. J Biol Chem 271:33425–33432. 8969205 10.1074/jbc.271.52.33425

[22] Keyhani NO, Roseman S 1996 The chitin catabolic cascade in the marine bacterium *Vibrio furnissii*. Molecular cloning, isolation, and characterization of a periplasmic chitodextrinase. J Biol Chem 271:33414–33424. 8969204 10.1074/jbc.271.52.33414

[23] Keyhani NO, Li XB, Roseman S 2000 Chitin catabolism in the marine bacterium *Vibrio furnissii*. Identification and molecular cloning of a chitoporin. J Biol Chem 275:33068–33076. 10913115 10.1074/jbc.M001041200

[24] Suginta W, Chumjan W, Mahendran KR, Janning P, Schulte A, Winterhalter M 2013 Molecular uptake of chitooligosaccharides through chitoporin from the marine bacterium *Vibrio harveyi*. PLoS One 8:e55126. doi:10.1371/journal.pone.0055126. 23383078 PMC3558487

[25] Suginta W, Chumjan W, Mahendran KR, Schulte A, Winterhalter M 2013 Chitoporin from *Vibrio harveyi*, a channel with exceptional sugar specificity. J Biol Chem 288:11038–11046. doi:10.1074/jbc.M113.454108. 23447539 PMC3630896

[26] Suzuki K, Uchiyama T, Suzuki M, Nikaidou N, Regue M, Watanabe T 2001 LysR-type transcriptional regulator ChiR is essential for production of all chitinases and a chitin-binding protein, CBP21, in *Serratia marcescens* 2170. Biosci Biotechnol Biochem 65:338–347. doi:10.1271/bbb.65.338. 11302167

[27] Toratani T, Suzuki K, Shimizu M, Sugimoto H, Watanabe T 2012 Regulation of chitinase production by the 5′-untranslated region of the *ybfM* in *Serratia marcescens* 2170. Biosci Biotechnol Biochem 76:1920–1924. doi:10.1271/bbb.120403. 23047109

[28] Overgaard M, Johansen J, Møller-Jensen J, Valentin-Hansen P 2009 Switching off small RNA regulation with trap-mRNA. Mol Microbiol 73:790–800. doi:10.1111/j.1365-2958.2009.06807.x. 19682266

[29] Figueroa-Bossi N, Valentini M, Malleret L, Fiorini F, Bossi L 2009 Caught at its own game: regulatory small RNA inactivated by an inducible transcript mimicking its target. Genes Dev 23:2004–2015. doi:10.1101/gad.541609. 19638370 PMC2751969

[30] Tsujibo H, Hatano N, Okamoto T, Endo H, Miyamoto K, Inamori Y 1999 Synthesis of chitinase in *Streptomyces thermoviolaceus* is regulated by a two-component sensor-regulator system. FEMS Microbiol Lett 181:83–90. doi:10.1111/j.1574-6968.1999.tb08829.x. 10564792

[31] Homerová D, Knirschová R, Kormanec J 2002 Response regulator ChiR regulates expression of chitinase gene, *chiC*, in *Streptomyces coelicolor*. Folia Microbiol (Praha) 47:499–505. doi:10.1007/BF02818788. 12503394

[32] Colson S, van Wezel GP, Craig M, Noens EEE, Nothaft H, Mommaas AM, Titgemeyer F, Joris B, Rigali S 2008 The chitobiose-binding protein, *DasA*, acts as a link between chitin utilization and morphogenesis in *Streptomyces coelicolor*. Microbiology 154:373–382. doi:10.1099/mic.0.2007/011940-0. 18227241

[33] Vaaje-Kolstad G, Bøhle LA, Gåseidnes S, Dalhus B, Bjørås M, Mathiesen G, Eijsink VGH 2012 Characterization of the chitinolytic machinery of *Enterococcus faecalis* V583 and high-resolution structure of its oxidative CBM33 enzyme. J Mol Biol 416:239–254. doi:10.1016/j.jmb.2011.12.033. 22210154

[34] Vaaje-Kolstad G, Bunaes AC, Mathiesen G, Eijsink VGH 2009 The chitinolytic system of *Lactococcus lactis* subsp. *lactis* comprises a nonprocessive chitinase and a chitin-binding protein that promotes the degradation of alpha- and beta-chitin. FEBS J 276:2402–2415. doi:10.1111/j.1742-4658.2009.06972.x. 19348025

[35] Watanabe T, Oyanagi W, Suzuki K, Tanaka H 1990 Chitinase system of *Bacillus circulans* WL-12 and importance of chitinase A1 in chitin degradation. J Bacteriol 172:4017–4022. doi:10.1128/jb.172.7.4017-4022.1990. 2361948 PMC213387

[36] Morimoto K, Karita S, Kimura T, Sakka K, Ohmiya K 1997 Cloning, sequencing, and expression of the gene encoding *Clostridium paraputrificum* chitinase ChiB and analysis of the functions of novel cadherin-like domains and a chitin-binding domain. J Bacteriol 179:7306–7314. doi:10.1128/jb.179.23.7306-7314.1997. 9393694 PMC179680

[37] Leisner JJ, Larsen MH, Jørgensen RL, Brøndsted L, Thomsen LE, Ingmer H 2008 Chitin hydrolysis by *Listeria* spp., including *L. monocytogenes*. Appl Environ Microbiol 74:3823–3830. doi:10.1128/AEM.02701-07. 18424542 PMC2446553

[38] Paspaliari DK, Loose JSM, Larsen MH, Vaaje-Kolstad G 2015 *Listeria monocytogenes* has a functional chitinolytic system and an active lytic polysaccharide monooxygenase. FEBS J 282:921–936. doi:10.1111/febs.13191. 25565565

[39] Leisner JJ, Larsen MH, Ingmer H, Petersen BO, Duus JØ, Palcic MM 2009 Cloning and comparison of phylogenetically related chitinases from *Listeria monocytogenes* EGD and *Enterococcus faecalis* V583. J Appl Microbiol 107:2080–2087. doi:10.1111/j.1365-2672.2009.04420.x. 19583793

[40] Frederiksen RF, Leisner JJ 2015 Effects of *Listeria monocytogenes* EGD-e and *Salmonella enterica* ser. Typhimurium LT2 chitinases on intracellular survival in *Dictyostelium discoideum* and mammalian cell lines. FEMS Microbiol Lett 362:fnv067. doi:10.1093/femsle/fnv067. 25908871

[41] Chaudhuri S, Bruno JC, Alonzo F, Xayarath B, Cianciotto NP, Freitag NE 2010 Contribution of chitinases to *Listeria monocytogenes* pathogenesis. Appl Environ Microbiol 76:7302–7305. doi:10.1128/AEM.01338-10. 20817810 PMC2976247

[42] Chaudhuri S, Gantner BN, Ye RD, Cianciotto NP, Freitag NE 2013 The *Listeria monocytogenes* ChiA chitinase enhances virulence through suppression of host innate immunity. mBio 4:e00617-12. doi:10.1128/mBio.00617-12. 23512964 PMC3604766

[43] Larsen MH, Leisner JJ, Ingmer H 2010 The chitinolytic activity of *Listeria monocytogenes* EGD is regulated by carbohydrates but also by the virulence regulator PrfA. Appl Environ Microbiol 76:6470–6476. doi:10.1128/AEM.00297-10. 20675445 PMC2950439

[44] Paspaliari DK, Mollerup MS, Kallipolitis BH, Ingmer H, Larsen MH 2014 Chitinase expression in *Listeria monocytogenes* is positively regulated by the *agr* system. PLoS One 9:e95385. doi:10.1371/journal.pone.0095385. 24752234 PMC3994053

[45] Nielsen JS, Larsen MH, Lillebæk EMS, Bergholz TM, Christiansen MHG, Boor KJ, Wiedmann M, Kallipolitis BH 2011 A small RNA controls expression of the chitinase ChiA in *Listeria monocytogenes*. PLoS One 6:e19019. doi:10.1371/journal.pone.0019019. 21533114 PMC3078929

[46] Holch A, Webb K, Lukjancenko O, Ussery D, Rosenthal BM, Gram L 2013 Genome sequencing identifies two nearly unchanged strains of persistent *Listeria monocytogenes* isolated at two different fish processing plants sampled 6 years apart. Appl Environ Microbiol 79:2944–2951. doi:10.1128/AEM.03715-12. 23435887 PMC3623136

[47] Popowska M, Markiewicz Z 2006 Characterization of *Listeria monocytogenes* protein Lmo0327 with murein hydrolase activity. Arch Microbiol 186:69–86. doi:10.1007/s00203-006-0122-8. 16763838

[48] Bierne H, Sabet C, Personnic N, Cossart P 2007 Internalins: a complex family of leucine-rich repeat-containing proteins in *Listeria monocytogenes*. Microbes Infect 9:1156–1166. doi:10.1016/j.micinf.2007.05.003. 17764999

[49] Sabet C, Lecuit M, Cabanes D, Cossart P, Bierne H 2005 LPXTG protein InlJ, a newly identified internalin involved in *Listeria monocytogenes* virulence. Infect Immun 73:6912–6922. doi:10.1128/IAI.73.10.6912-6922.2005. 16177371 PMC1230919

[50] Kazmierczak MJ, Mithoe SC, Boor KJ, Wiedmann M 2003 *Listeria monocytogenes* sigma B regulates stress response and virulence functions. J Bacteriol 185:5722–5734. doi:10.1128/JB.185.19.5722-5734.2003. 13129943 PMC193959

[51] Arous S, Buchrieser C, Folio P, Glaser P, Namane A, Hébraud M, Héchard Y 2004 Global analysis of gene expression in an *rpoN* mutant of *Listeria monocytogenes*. Microbiology 150:1581–1590. doi:10.1099/mic.0.26860-0. 15133119

[52] McGann P, Ivanek R, Wiedmann M, Boor KJ 2007 Temperature-dependent expression of *Listeria monocytogenes* internalin and internalin-like genes suggests functional diversity of these proteins among the listeriae. Appl Environ Microbiol 73:2806–2814. doi:10.1128/AEM.02923-06. 17337561 PMC1892884

[53] Bierne H, Cossart P 2007 *Listeria monocytogenes* surface proteins: from genome predictions to function. Microbiol Mol Biol Rev 71:377–397. doi:10.1128/MMBR.00039-06. 17554049 PMC1899877

[54] Schubert WD, Göbel G, Diepholz M, Darji A, Kloer D, Hain T, Chakraborty T, Wehland J, Domann E, Heinz DW 2001 Internalins from the human pathogen *Listeria monocytogenes* combine three distinct folds into a contiguous internalin domain. J Mol Biol 312:783–794. doi:10.1006/jmbi.2001.4989. 11575932

[55] Schauer K, Geginat G, Liang C, Goebel W, Dandekar T, Fuchs TM 2010 Deciphering the intracellular metabolism of *Listeria monocytogenes* by mutant screening and modelling. BMC Genomics 11:573. doi:10.1186/1471-2164-11-573. 20955543 PMC3091722

[56] García-del Portillo F, Calvo E, D'Orazio V, Pucciarelli MG 2011 Association of ActA to peptidoglycan revealed by cell wall proteomics of intracellular *Listeria monocytogenes*. J Biol Chem 286:34675–34689. doi:10.1074/jbc.M111.230441. 21846725 PMC3186376

[57] Garmyn D, Augagneur Y, Gal L, Vivant A-L, Piveteau P 2012 *Listeria monocytogenes* differential transcriptome analysis reveals temperature-dependent Agr regulation and suggests overlaps with other regulons. PLoS One 7:e43154. doi:10.1371/journal.pone.0043154. 23024744 PMC3443086

[58] McGann P, Raengpradub S, Ivanek R, Wiedmann M, Boor KJ 2008 Differential regulation of *Listeria monocytogenes* internalin and internalin-like genes by sigma B and PrfA as revealed by subgenomic microarray analyses. Foodborne Pathog Dis 5:417–435. doi:10.1089/fpd.2008.0085. 18713061 PMC2688707

[59] Milohanic E, Glaser P, Coppée J-Y, Frangeul L, Vega Y, Vázquez-Boland JA, Kunst F, Cossart P, Buchrieser C 2003 Transcriptome analysis of *Listeria monocytogenes* identifies three groups of genes differently regulated by PrfA. Mol Microbiol 47:1613–1625. doi:10.1046/j.1365-2958.2003.03413.x. 12622816

[60] Mujahid S, Orsi RH, Vangay P, Boor KJ, Wiedmann M 2013 Refinement of the *Listeria monocytogenes* σ^B^ regulon through quantitative proteomic analysis. Microbiology 159:1109–1119. doi:10.1099/mic.0.066001-0. 23618998 PMC3709693

[61] Glaser P, Frangeul L, Buchrieser C, Rusniok C, Amend A, Baquero F, Berche P, Bloecker H, Brandt P, Chakraborty T, Charbit A, Chetouani F, Couvé E, de Daruvar A, Dehoux P, Domann E, Domínguez-Bernal G, Duchaud E, Durant L, Dussurget O, Entian KD, Fsihi H, García-del Portillo F, Garrido P, Gautier L, Goebel W, Gómez-López N, Hain T, Hauf J, Jackson D, Jones LM, Kaerst U, Kreft J, Kuhn M, Kunst F, Kurapkat G, Madueno E, Maitournam A, Vicente JM, Ng E, Nedjari H, Nordsiek G, Novella S, de Pablos B, Pérez-Diaz JC, Purcell R, Remmel B, Rose M, Schlueter T, Simoes N, Tierrez A, Vázquez-Boland JA, Voss H, Wehland J, Cossart P 2001 Comparative genomics of *Listeria* species. Science 294:849–852. 11679669 10.1126/science.1063447

[62] Wulff G, Gram L, Ahrens P, Vogel BF 2006 One group of genetically similar *Listeria monocytogenes* strains frequently dominates and persists in several fish slaughter- and smokehouses. Appl Environ Microbiol 72:4313–4322. doi:10.1128/AEM.02288-05. 16751546 PMC1489582

[63] Cao M, Bitar A, Marquis H 2007 A *mariner*-based transposition system for *Listeria monocytogenes*. Appl Environ Microbiol 73:2758–2761. doi:10.1128/AEM.02844-06. 17308180 PMC1855599

[64] Kastbjerg VG, Larsen MH, Gram L, Ingmer H 2010 Influence of sublethal concentrations of common disinfectants on expression of virulence genes in *Listeria monocytogenes*. Appl Environ Microbiol 76:303–309. doi:10.1128/AEM.00925-09. 19897753 PMC2798637

