# Supplementary material for: Chitinase Expression in Listeria monocytogenes Is Influenced by lmo0327, Which Encodes an Internalin-Like Protein
Source: Appl Environ Microbiol. 2017 Oct 31;83(22):e01283-17. doi: 10.1128/AEM.01283-17 (PMC5666140; doi:10.1128/AEM.01283-17)
Supplement: Supplemental material [file supp_83_22_e01283-17__index.html]

Supplemental material 

# Chitinase Expression in Listeria monocytogenes Is Influenced by *lmo0327*, Which Encodes an Internalin-Like Protein

## Supplemental material

- Supplemental file 1 -

  Growth curves of wild-type EGD and a mutant strain with insertional inactivation of *lmo0327* (Fig. S1); nucleotide alignment of the *lmo0327* loci of strains N53-1 and EGD-e (Fig. S2); protein alignment of the predicted Lmo0327 sequences of strains N53-1 and EGD-e (Fig. S3).

  PDF, 1.7M
