# Supplementary material for: Chitinase Expression in Listeria monocytogenes Is Influenced by lmo0327, Which Encodes an Internalin-Like Protein
Source: Appl Environ Microbiol. 2017 Oct 31;83(22):e01283-17. doi: 10.1128/AEM.01283-17 (PMC5666140; doi:10.1128/AEM.01283-17)
Supplement: Supplemental material [file AEM.01283-17_zam999118139s1.pdf]

SUPPLEMENTAL MATERIAL

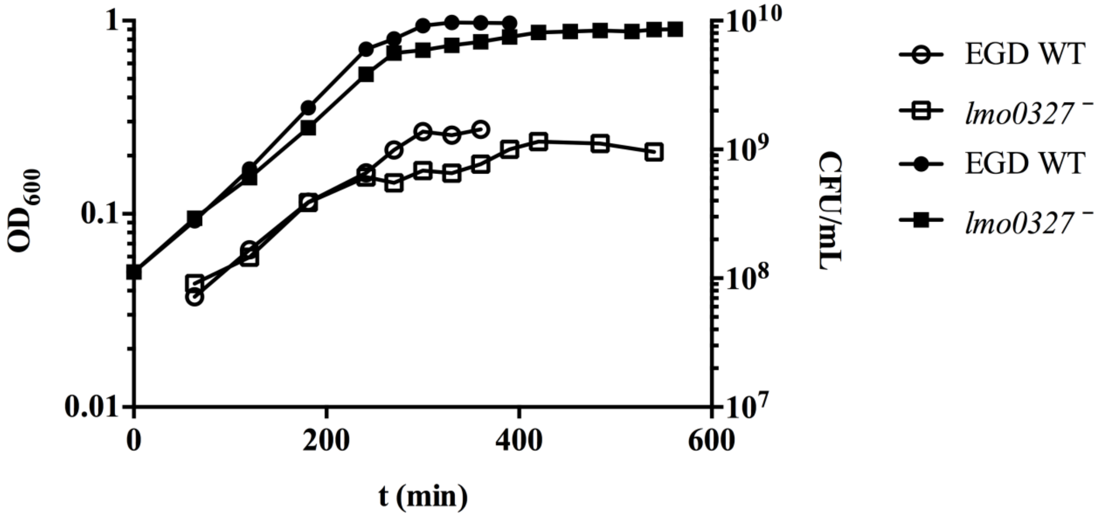

Figure S1. Growth curves of wild-type EGD and a mutant strain with insertional inactivation of *lmo0327*. The strains were grown at 30°C in LB supplemented with 0.05% glucose, and the OD_{600} (filled symbols), and CFU/mL (open symbols) were measured at selected time points.

```
                                  20                                  40                                  60                                  80
N53-1          ATTTTAAAGA AAATATTTAT TGCATTAATT GCGCTTGTAG TTATTATCAA TACAGTTAGT TTTACGGCTG TTTCTGCAAG 80
EGD-e lmo0327  attttaaaga aaatatttat tgcattaatt gcgcttgtag ttattatcaa tacagttagt tttacgtctg tttctgcaag 80
Consensus      ATTTTAAAGA AAATATTTAT TGCATTAATT GCGCTTGTAG TTATTATCAA TACAGTTAGT TTTACGNCTG TTTCTGCAAG
Conservation   100% 0%

                                 100                                 120                                 140                                 160
N53-1          CACACCTACA TGGTTAGAAC AAACCATGAA TAATAATGAA CCGTTTATAA AAGAGATTGA AAAAGAAACT GGTAAGACGA 160
EGD-e lmo0327  cacacctaca tggttagaac aaaccatgaa taataatgaa ccgtttataa aagagattga aactgaaact ggtaagacga 160
Consensus      CACACCTACA TGGTTAGAAC AAACCATGAA TAATAATGAA CCGTTTATAA AAGAGATTGA AANNGAAACT GGTAAGACGA
Conservation   100% 0%

                                 180                                 200                                 220                                 240
N53-1          GAGCGAATAT TACACAGACG GATTTAGAAG CTATAACCGC CTTGCGAGTA ACAGGGGCAA GTGATATACC GACTAATATC 240
EGD-e lmo0327  gagcgaatat tacacagaca gatttagaag caataactgc cttgcgagta acaggggcaa gtgatatccc ggcaaatatc 240
Consensus      GAGCGAATAT TACACAGACN GATTTAGAAG CNATAACNGC CTTGCGAGTA ACAGGGGCAA GTGATATNCC GNCNAATATC
Conservation   100% 0%

                                 260                                 280                                 300                                 320
N53-1          GATATGTTAA CTCATTTAAC TACGTTAGAA GTTAAAAATG GAACATTATC TTCCGTATCT AACTCAGTGG GGAATTTAAA 320
EGD-e lmo0327  gatatgttaa ctcatttaac cctgttaaaa gcggtacaag ggacgatatc ttcagtgcct aactctgtgg gtaatttaaa 320
Consensus      GATATGTTAA CTCATTTAAC NNNGTTANAA GNNNNANANG GNACNNTATC TTCNGTNNCT AACTCNGTGG GNAATTTAAA
Conservation   100% 0%

                                 340                                 360                                 380                                 400
N53-1          GGAGTTAGAA ATCTTACTTC TGAATGATAA TAATTTAAGC ACCTTTCCAA TGATTGTTTT CCAACTACCT AAGTTAGAAG 400
EGD-e lmo0327  agagctaaaa atcttaaatc ttaatactaa tcatttaagt aattttccga cgattgttta tcaactgcct aagttagaag 400
Consensus      NGAGNTANAA ATCTTANNTC TNAATNNTAA TNATTTAAGN ANNTTTCCNA NGATTGTTTN NCAACTNCCT AAGTTAGAAG
Conservation   100% 0%

                                 420                                 440                                 460                                 480
N53-1          AATTACAAGT TTCTGGGGGA ACAATAGAAG AAATCCCTGC CACAATAACA AATATGGCGA GTCATTTGAA ATTCTTAGGG 480
EGD-e lmo0327  aattacaact taatgatgga acgattgaag agattcctgc aacaataacg aatatggcga gccatttaaa gtttttaact 480
Consensus      AATTACAANT TNNTGNNGGA ACNATNGAAG ANATNCCTGC NACAATAACN AATATGGCGA GNCATTTNAA NTTNTTANNN
Conservation   100% 0%

                                 500                                 520                                 540                                 560
N53-1          ATATTAAATA ACCATTTAGT GAAGGTTCCA GACGCAATTT TCACTACGAA CTGGACAAAC TCATCTACGG GAGACTTAGA 560
EGD-e lmo0327  gtaaataata accgcttagt gaaagttcca actataatat tctcaacgaa ttggtcaaac tcatctacgg gagaattaga 560
Consensus      NTANNNAATA ACCNNTTAGT GAANGTTCCA NNNNNAATNT TCNCNACGAA NTGGNCAAAC TCATCTACGG GAGANTTAGA
Conservation   100% 0%

                                 580                                 600                                 620                                 640
N53-1          CTTAATGGCT GCAGGGAACC AGATTGTAAC CAATATTCCT GCCAACTATA TAAGCCAGTT TAATAATGGG AATAACCTAT 640
EGD-e lmo0327  tttatttacc actggaaatc agattgtaac ggatattcct gccaactatg taagtaactt taataatgga caaaacatgt 640
Consensus      NTTANTNNCN NCNGGNAANC AGATTGTAAC NNATATTCCT GCCAACTATN TAAGNNANTT TAATAATGGN NANAACNTNT
Conservation   100% 0%

                                 660                                 680                                 700                                 720
N53-1          TAGAATTTTA CGATAACAAC TATCAAAAAC AAGACCAACT AACTACAACA CCTGGATATA CCATTGATGT ACCTGTTGGA 720
EGD-e lmo0327  tagaatttta cgatggtcaa tttcaaaaac aagaccaact aactacaaca cctggatata ccattgatgt acctgttgga 720
Consensus      TAGAATTTTA CGATNNNNAN TNTCAAAAAC AAGACCAACT AACTACAACA CCTGGATATA CCATTGATGT ACCTGTTGGA
Conservation   100% 0%

                                 740                                 760                                 780                                 800
N53-1          ACTGATTTTA ATCAATTAAC ACCAGACAAA ACGAAACTCG CACTAACATC TGGACGAACT TTACTCGCGC AACACGAATT 800
EGD-e lmo0327  actgatttta atcaattaac accagacaaa acgaaactcg cactaacatc tggacgaact ttactcgcgc aacacgaatt 800
Consensus      ACTGATTTTA ATCAATTAAC ACCAGACAAA ACGAAACTCG CACTAACATC TGGACGAACT TTACTCGCGC AACACGAATT
Conservation   100% 0%

                                 820                                 840                                 860                                 880
N53-1          CGAGTATTAT GATGATGGTT CAAGTTCACT AATCCACAAT GGCGTGGCAG CAGCACCGGG TCAAGCGACC ATCTTCATCA 880
EGD-e lmo0327  cgagtattat gatgatggtt caagttcact aatccacaat ggcgtggcag cagcaccggg tcaagcgacc attttcatca 880
Consensus      CGAGTATTAT GATGATGGTT CAAGTTCACT AATCCACAAT GGCGTGGCAG CAGCACCGGG TCAAGCGACC ATNTTCATCA
Conservation   100% 0%

                                 900                                 920                                 940                                 960
N53-1          AGAGCAAGTT TTCCACGCAA TCGAACAAAT TTGCCAGAAC ACAAGTTACA GTAAACATCA CCGCGCTAAA CGGCGGACCT 960
EGD-e lmo0327  aaagcaagtt ttccacgcaa tcgaacaaat ttgccagaac acaagttaca gtaaacatcg ctgcgctaaa cggcggacct 960
Consensus      ANAGCAAGTT TTCCACGCAA TCGAACAAAT TTGCCAGAAC ACAAGTTACA GTAAACATCN CNGCGCTAAA CGGCGGACCT
Conservation   100% 0%
```

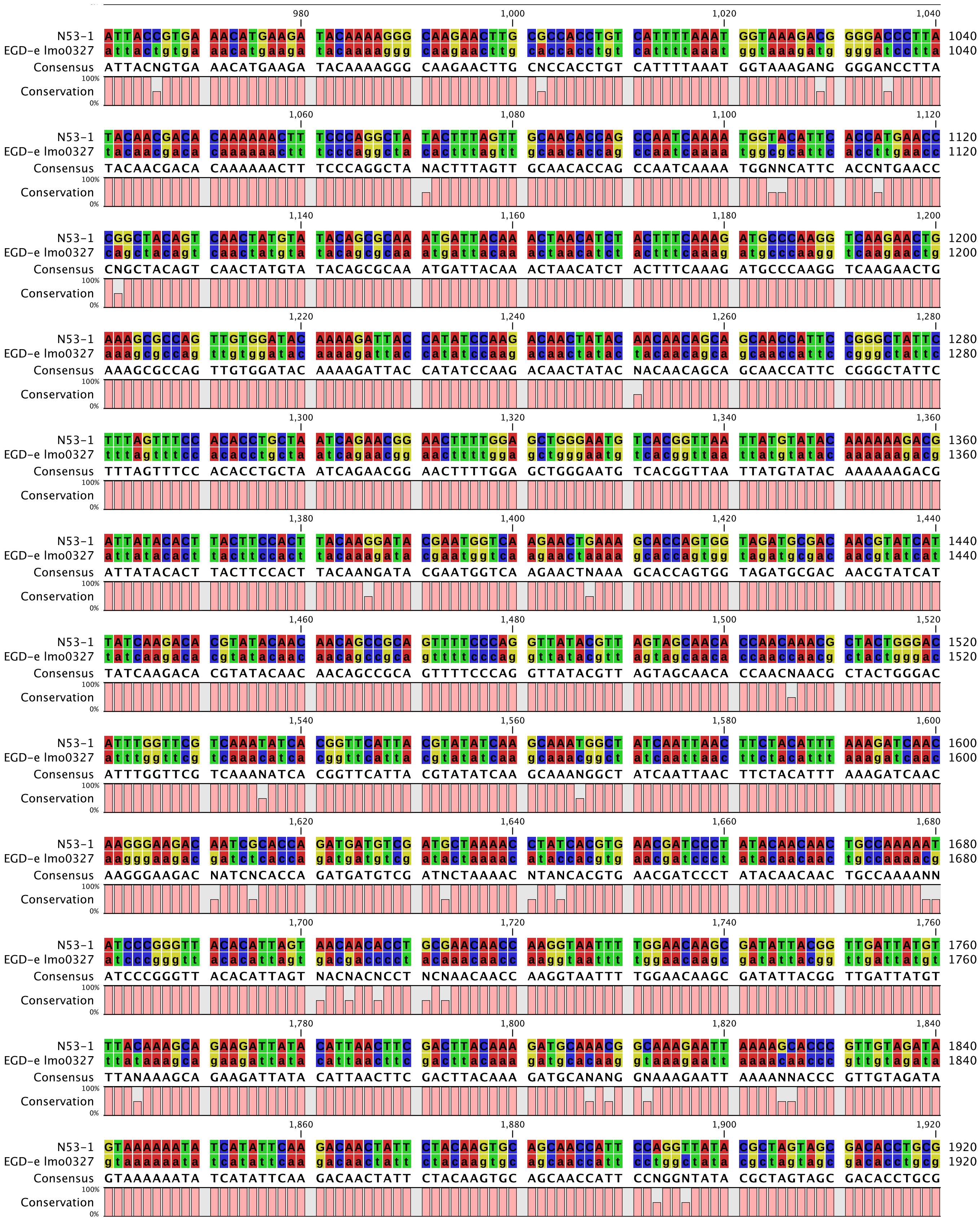
980 1,000 1,020 1,040
N53-1 ATTACCGTGA AACATGAAGA TACAAAAGGG CAAGAACTTG CGCCACCTGT CATTTTAAAT GGTAAAGACG GGGACCCTTA 1040
EGD-e lmo0327 attactgtga aacatgaaga tacaaaaggg caagaacttg caccacctgt cattttaaat ggtaaagatg gggatcctta 1040
Consensus ATTACNGTGA AACATGAAGA TACAAAAGGG CAAGAACTTG CNCCACCTGT CATTTTAAAT GGTAAAGANG GGGANCCTTA
Conservation 100% 0%
1,060 1,080 1,100 1,120
N53-1 TACAACGACA CAAAAAACTT TCCCAGGCTA TACTTTAGTT GCAACACCAG CCAATCAAAA TGGTACATTC ACCATGAACC 1120
EGD-e lmo0327 tacaacgaca caaaaaactt tcccaggcta cactttagtt gcaacaccag ccaatcaaaa tggcgcattc accttgaacc 1120
Consensus TACAACGACA CAAAAAACTT TCCCAGGCTA NACTTTAGTT GCAACACCAG CCAATCAAAA TGGNNCATTC ACCNTGAACC
Conservation 100% 0%
1,140 1,160 1,180 1,200
N53-1 CGGCTACAGT CAACTATGTA TACAGCGCAA ATGATTACAA ACTAACATCT ACTTTCAAAG ATGCCCAAGG TCAAGAACTG 1200
EGD-e lmo0327 cagctacagt caactatgta tacagcgcaa atgattacaa actaacatct actttcaaag atgcccaagg tcaagaactg 1200
Consensus CNGCTACAGT CAACTATGTA TACAGCGCAA ATGATTACAA ACTAACATCT ACTTTCAAAG ATGCCCAAGG TCAAGAACTG
Conservation 100% 0%
1,220 1,240 1,260 1,280
N53-1 AAAGCGCCAG TTGTGGATAC AAAAGATTAC CATATCCAAG ACAACTATAC AACAACAGCA GCAACCATTC CGGGCTATTC 1280
EGD-e lmo0327 aaagcgccag ttgtggatac aaaagattac catatccaag acaactatac tacaacagca gcaaccattc cgggctattc 1280
Consensus AAAGCGCCAG TTGTGGATAC AAAAGATTAC CATATCCAAG ACAACTATAC NACAACAGCA GCAACCATTC CGGGCTATTC
Conservation 100% 0%
1,300 1,320 1,340 1,360
N53-1 TTTAGTTTCC ACACCTGCTA ATCAGAACGG AACTTTTGGA GCTGGGAATG TCACGGTTAA TTATGTATAC AAAAAAGACG 1360
EGD-e lmo0327 tttagtttcc acacctgcta atcagaacgg aacttttgga gctgggaatg tcacggttaa ttatgtatac aaaaaagacg 1360
Consensus TTTAGTTTCC ACACCTGCTA ATCAGAACGG AACTTTTGGA GCTGGGAATG TCACGGTTAA TTATGTATAC AAAAAAGACG
Conservation 100% 0%
1,380 1,400 1,420 1,440
N53-1 ATTATACACT TACTTCCACT TACAAGGATA CGAATGGTCA AGAACTGAAA GCACCAGTGG TAGATGCGAC AACGTATCAT 1440
EGD-e lmo0327 attatacact tacttccact tacaaagata cgaatggtca agaactaaaa gcaccagtgg tagatgcgac aacgtatcat 1440
Consensus ATTATACACT TACTTCCACT TACAANGATA CGAATGGTCA AGAACTNAAA GCACCAGTGG TAGATGCGAC AACGTATCAT
Conservation 100% 0%
1,460 1,480 1,500 1,520
N53-1 TATCAAGACA CGTATACAAC AACAGCCGCA GTTTTCCCAG GTTATACGTT AGTAGCAACA CCAACAAACG CTACTGGGAC 1520
EGD-e lmo0327 tatcaagaca cgtatacaac aacagccgca gttttcccag gttatacgtt agtagcaaca ccaaccaacg ctactgggac 1520
Consensus TATCAAGACA CGTATACAAC AACAGCCGCA GTTTTCCCAG GTTATACGTT AGTAGCAACA CCAACNAACG CTACTGGGAC
Conservation 100% 0%
1,540 1,560 1,580 1,600
N53-1 ATTTGGTTCG TCAAATATCA CGGTTCATTA CGTATATCAA GCAAATGGCT ATCAATTAAC TTCTACATTT AAAGATCAAC 1600
EGD-e lmo0327 atttggttcg tcaaacatca cggttcatta cgtatatcaa gcaaacggct atcaattaac ttctacattt aaagatcaac 1600
Consensus ATTTGGTTCG TCAAANATCA CGGTTCATTA CGTATATCAA GCAAANGGCT ATCAATTAAC TTCTACATTT AAAGATCAAC
Conservation 100% 0%
1,620 1,640 1,660 1,680
N53-1 AAGGGAAGAC AATCGCACCA GATGATGTCG ATGCTAAAAC CTATCACGTG AACGATCCCT ATACAACAAC TGCCAAAAAT 1680
EGD-e lmo0327 aagggaagac gatctcacca gatgatgtcg atactaaaac ataccacgtg aacgatccct atacaacaac tgccaaaacg 1680
Consensus AAGGGAAGAC NATCNCACCA GATGATGTCG ATNCTAAAAC NTANCACGTG AACGATCCCT ATACAACAAC TGCCAAAANN
Conservation 100% 0%
1,700 1,720 1,740 1,760
N53-1 ATCCCGGGTT ACACATTAGT AACAACACCT GCGAACAACC AAGGTAATTT TGGAACAAGC GATATTACGG TTGATTATGT 1760
EGD-e lmo0327 atcccgggtt acacattagt gacgacccct acaaacaacc aaggtaattt tggaacaagc gatattacgg ttgattatgt 1760
Consensus ATCCCGGGTT ACACATTAGT NACNACNCCT NCNAACAACC AAGGTAATTT TGGAACAAGC GATATTACGG TTGATTATGT
Conservation 100% 0%
1,780 1,800 1,820 1,840
N53-1 TTACAAAGCA GAAGATTATA CATTAACTTC GACTTACAAA GATGCAAACG GCAAAGAATT AAAAGCACCC GTTGTAGATA 1840
EGD-e lmo0327 ttataaagca gaagattata cattaacttc gacttacaaa gatgcacaag gtaaagaatt aaaacaaccc gttgtagata 1840
Consensus TTANAAAGCA GAAGATTATA CATTAACTTC GACTTACAAA GATGCANANG GNAAAGAATT AAAANNACCC GTTGTAGATA
Conservation 100% 0%
1,860 1,880 1,900 1,920
N53-1 GTAAAAAATA TCATATTCAA GACAACTATT CTACAAGTGC AGCAACCATT CCAGGTTATA CGCTAGTAGC GACACCTGCG 1920
EGD-e lmo0327 gtaaaaaata tcatattcaa gacaactatt ctacaagtgc agcaaccatt cctggctata cgctagtagc gacacctgcg 1920
Consensus GTAAAAAATA TCATATTCAA GACAACTATT CTACAAGTGC AGCAACCATT CCNGGNTATA CGCTAGTAGC GACACCTGCG
Conservation 100% 0%

```
                     1,940                 1,960                 1,980                 2,000
N53-1          AACGAAACAG GAACCTTCCA TACAAGCGAT GTCACGGTTA ATTATGTGTA TAAATTAACA GACTTAAAAT TAACTTCGAC 2000
EGD-e lmo0327  aacgaaacag gaaccttcca tacaagcgat gtcacggtta attatgtgta taaattaaca gacttaaaat taacttcgac 2000
Consensus      AACGAAACAG GAACCTTCCA TACAAGCGAT GTCACGGTTA ATTATGTGTA TAAATTAACA GACTTAAAAT TAACTTCGAC
Conservation   100% 0%

                     2,020                 2,040                 2,060                 2,080
N53-1          ATATAAAGAT GCGCAAGGAA CTGAGTTAAA ACCACCCGTT GTAGATAGCA AAACGTATCA TATTCAAGAT AACTATGCTA 2080
EGD-e lmo0327  atataaagat gcgcaaggaa cggagttaaa accacccgtt gtagatagca aaacgtatca tattcaagat aactatgcta 2080
Consensus      ATATAAAGAT GCGCAAGGAA CNGAGTTAAA ACCACCCGTT GTAGATAGCA AAACGTATCA TATTCAAGAT AACTATGCTA
Conservation   100% 0%

                     2,100                 2,120                 2,140                 2,160
N53-1          CAACTGCAGC TGTCATTCCT GGCTATACAT TAGTCGCAAC ACCAGCGAAT CAATCCGGTA CATTTGGC-- ---------- 2148
EGD-e lmo0327  caactgcagc cgtgatcccg ggctacacat tagtggcaac accagcgaac caatcaggca cttttggtag cacggatatc 2160
Consensus      CAACTGCAGC NGTNATNCCN GGCTANACAT TAGTNGCAAC ACCAGCGAAN CAATCNGGNA CNTTTGGNAG CACGGATATC
Conservation   100% 0%

                     2,180                 2,200                 2,220                 2,240
N53-1          ---------- ---------- ---------- ---------- ---------- ---------- ---------- ---------- 2148
EGD-e lmo0327  caagtgaact atgtgtacca agcagttgct tataaactaa cttcgacgta taaagatcaa caagggaatg atttagcttt 2240
Consensus      CAAGTGAACT ATGTGTACCA AGCAGTTGCT TATAAACTAA CTTCGACGTA TAAAGATCAA CAAGGGAATG ATTTAGCTTT
Conservation   100% 0%

                     2,260                 2,280                 2,300                 2,320
N53-1          ---------- ---------- ---------- ---------- ---------- ---------- ---------- ---------- 2148
EGD-e lmo0327  accaaaagta gatacaaaaa cgtaccatat tcaggatggt tacacgacga gtgatattgc tattccaggc tatacattag 2320
Consensus      ACCAAAAGTA GATACAAAAA CGTACCATAT TCAGGATGGT TACACGACGA GTGATATTGC TATTCCAGGC TATACATTAG
Conservation   100% 0%

                     2,340                 2,360                 2,380                 2,400
N53-1          ---------- ---------- ---------- ---------- ---------- ---------- ---------- ---------- 2148
EGD-e lmo0327  tcgcggctcc aacgaatcaa acaggtacat ttggtgcgag tgatgtaacc gtgaattatg tctacaaagc caatgactac 2400
Consensus      TCGCGGCTCC AACGAATCAA ACAGGTACAT TTGGTGCGAG TGATGTAACC GTGAATTATG TCTACAAAGC CAATGACTAC
Conservation   100% 0%

                     2,420                 2,440                 2,460                 2,480
N53-1          ---------- ---------- ---------- ---------- ---------- ---------- ---------- ---------- 2148
EGD-e lmo0327  acactaactt caacatataa agatgcacaa ggtaaggaat taaaaacgcc agtcatagac agtcaaaaat atcacattaa 2480
Consensus      ACACTAACTT CAACATATAA AGATGCACAA GGTAAGGAAT TAAAAACGCC AGTCATAGAC AGTCAAAAAT ATCACATTAA
Conservation   100% 0%

                     2,500                 2,520                 2,540                 2,560
N53-1          ---------- ---------- ---------- ---------- ---------- ---------- ---------- ---------- 2148
EGD-e lmo0327  cgacacttat actacgacag gagctactat tccaggttac acattagtcg cagcaccagc gaatcaatca ggtacatttg 2560
Consensus      CGACACTTAT ACTACGACAG GAGCTACTAT TCCAGGTTAC ACATTAGTCG CAGCACCAGC GAATCAATCA GGTACATTTG
Conservation   100% 0%

                     2,580                 2,600                 2,620                 2,640
N53-1          --GCGGCGAA TGTAACAGTC AACTATGTAT ACAAAGCAGA TGATTACACA TTAACTTCGA CCTACAAAGA TGTAAACGGC 2226
EGD-e lmo0327  gcgcggcgaa tgtaacagtc aactatgtat acaaagcaga tgattacaca ttaacttcga cctacaaaga tgcaaacggc 2640
Consensus      GCGCGGCGAA TGTAACAGTC AACTATGTAT ACAAAGCAGA TGATTACACA TTAACTTCGA CCTACAAAGA TGNAAACGGC
Conservation   100% 0%

                     2,660                 2,680                 2,700                 2,720
N53-1          AAAGAATTAA AAGCGCCAGT TGTAGACAGC AAAACTTACC ATACAAAAGA TAACTATTCT ACTAGCGCAG CAACCATTCC 2306
EGD-e lmo0327  aaagaattaa aagcgccagt tgtagacagc aaaacttacc atacaaaaga taactattct actagcgcag caaccattcc 2720
Consensus      AAAGAATTAA AAGCGCCAGT TGTAGACAGC AAAACTTACC ATACAAAAGA TAACTATTCT ACTAGCGCAG CAACCATTCC
Conservation   100% 0%

                     2,740                 2,760                 2,780                 2,800
N53-1          AGGCTACACA CTAGTGGCAG CACCAGCAAA TCAAACAGGT ACATTTAATA CGAGCAATGT GACAGTCAAT TATGTTTACA 2386
EGD-e lmo0327  tggctacaca ctagtggcag caccagcaaa tcaaacaggt acatttaata cgagcaatgt gacagtcaat tatttttaca 2800
Consensus      NGGCTACACA CTAGTGGCAG CACCAGCAAA TCAAACAGGT ACATTTAATA CGAGCAATGT GACAGTCAAT TATNTTTACA
Conservation   100% 0%

                     2,820                 2,840                 2,860                 2,880
N53-1          AAGCGAATGA ATATACCTTA ACTGCCACTT TCAAAAATGT ACAAGGAACA GAACTAAAAC CAGCTATCGT GAAAAAAGGA 2466
EGD-e lmo0327  aagcgaatga atatacctta acttccactt tcaaaaatgt acaaggaaca gaactaaaac cagctatcgt gaaaaaagga 2880
Consensus      AAGCGAATGA ATATACCTTA ACTNCCACTT TCAAAAATGT ACAAGGAACA GAACTAAAAC CAGCTATCGT GAAAAAAGGA
Conservation   100% 0%
```

```
                                          2,900                                      2,920                                      2,940                                      2,960
N53-1          TTTATTATTA AGGATGGCTA TGCGACTAGT GGCGTAACAA TTCCAGGCTA TACATTAGTG GCAACACCAT CGAACAAAAA 2546
EGD-e lmo0327  tttattatta aggatggcta ttcgactagt ggcgtaacaa ttccaggcta tacattagtt gcaacaccat cgaacaaaaa 2960
Consensus      TTTATTATTA AGGATGGCTA TNCGACTAGT GGCGTAACAA TTCCAGGCTA TACATTAGTN GCAACACCAT CGAACAAAAA
Conservation

                                          2,980                                      3,000                                      3,020                                      3,040
N53-1          AGGAACATTT GGTGCTAGTA ATGTAACAGT TAATTATGTA TATAAAGCGA ATGGTTATGC ATTAATCACA ACATATAAAG 2626
EGD-e lmo0327  aggaacattt ggcgctagta atgtaacagt taattatgta tataaagcga atggttatgc attaatcaca acatataaag 3040
Consensus      AGGAACATTT GGNGCTAGTA ATGTAACAGT TAATTATGTA TATAAAGCGA ATGGTTATGC ATTAATCACA ACATATAAAG
Conservation

                                          3,060                                      3,080                                      3,100                                      3,120
N53-1          ACACACAAGG TAAAGACTTA AAACCACTAG CGATTGATAC AAAAACATAC AATATTAACG ACCCATACAC TGCAACAGCG 2706
EGD-e lmo0327  acacacaagg taaagactta aaaccactag ccattgatac aaaaacatac aatattaacg acccatacac tgcaacagct 3120
Consensus      ACACACAAGG TAAAGACTTA AAACCACTAG CNATTGATAC AAAAACATAC AATATTAACG ACCCATACAC TGCAACAGCN
Conservation

                                          3,140                                      3,160                                      3,180                                      3,200
N53-1          CTGAACATCC CAGGTTACAC ATTAACAACT ACACCAGCCA ATGAAAAAGG TGTATTTGGC GCAAGTGATG AAACAGTTAA 2786
EGD-e lmo0327  ctgaacatcc caggctacac attaacaact acaccagcca atgaaaaagg tgtatttggc gcaagtgatg aaacagttaa 3200
Consensus      CTGAACATCC CAGGNTACAC ATTAACAACT ACACCAGCCA ATGAAAAAGG TGTATTTGGC GCAAGTGATG AAACAGTTAA
Conservation

                                          3,220                                      3,240                                      3,260                                      3,280
N53-1          TTACGTATAT AAAGCAAATG ATTATACGTT AACAACTACC TACAAAGACG CAAACGGCAA AGAACTACAA GCACCTAAAG 2866
EGD-e lmo0327  ttacgtatat aaagcaaatg attatacgtt aacaactacc tacaaagacg caaacggcaa agaactacaa gcacctaaag 3280
Consensus      TTACGTATAT AAAGCAAATG ATTATACGTT AACAACTACC TACAAAGACG CAAACGGCAA AGAACTACAA GCACCTAAAG
Conservation

                                          3,300                                      3,320                                      3,340                                      3,360
N53-1          TAGACGCGAA AACTTATCAT ATCCAAGACA CATACAAAAC AACTGCAGCA GTCATTCCAG GCTATACATT AGTCGCAACA 2946
EGD-e lmo0327  tagacgcgaa aacttatcat atccaagaca catacaaaac aactgcagca gtcattcctg gatatacatt agtcgcaata 3360
Consensus      TAGACGCGAA AACTTATCAT ATCCAAGACA CATACAAAAC AACTGCAGCA GTCATTCCNG GNTATACATT AGTCGCAANA
Conservation

                                          3,380                                      3,400                                      3,420                                      3,440
N53-1          CCTAAGAATG ACCAAGGTAC TTTTGGAGCG AACAATGTAA CTGTGAATTA CGTTTACCAA ACAAATGATT ACACGCTAAC 3026
EGD-e lmo0327  cctaagaatg accaaggtac ttttggagcg aacaatgtaa ctgtgaatta cgtgtaccaa acaaatgatt acacgctaac 3440
Consensus      CCTAAGAATG ACCAAGGTAC TTTTGGAGCG AACAATGTAA CTGTGAATTA CGTNTACCAA ACAAATGATT ACACGCTAAC
Conservation

                                          3,460                                      3,480                                      3,500                                      3,520
N53-1          TTCAACGTTT AAAGACACAC AAGGAAACGA ATTAAAAGCA GCAGAAGTAG ACGCACAAAA ATACCATATT CATGACACTT 3106
EGD-e lmo0327  tgcgacattt aaagatgctc aaggaaacga attaaaagca acagaagtag acgcacaaaa ataccatatt catgacactt 3520
Consensus      TNCNACNTTT AAAGANNCNC AAGGAAACGA ATTAAAAGCA NCAGAAGTAG ACGCACAAAA ATACCATATT CATGACACTT
Conservation

                                          3,540                                      3,560                                      3,580                                      3,600
N53-1          ACACATCGAA AGTAGCCGTG ATTCCGGGTT ACACTTTAGA AAAAACACCA ACGAACGAAA CGGGTACTTT TAATGCGAGT 3186
EGD-e lmo0327  acacatcgaa agcagccgtg attccaggtt acactttaga aaaaacacca acgaacgaaa caggtacttt taatgcaagt 3600
Consensus      ACACATCGAA AGNAGCCGTG ATTCCNGGTT ACACTTTAGA AAAAACACCA ACGAACGAAA CNGGTACTTT TAATGCNAGT
Conservation

                                          3,620                                      3,640                                      3,660                                      3,680
N53-1          GATATTCAAG TGAATTATGT GTATAAAGCA AATGATTATC AACTTACTTC TACTTTTAAA GATCAACAAG GCAACGAAAT 3266
EGD-e lmo0327  gatattcaag taaattatgt gtataaagca aatgattatc aacttacttc tacgtttaaa gatcaacaag gcaacgaaat 3680
Consensus      GATATTCAAG TNAATTATGT GTATAAAGCA AATGATTATC AACTTACTTC TACNTTTAAA GATCAACAAG GCAACGAAAT
Conservation

                                          3,700                                      3,720                                      3,740                                      3,760
N53-1          AGCATTACCA ACAGTTGATG CAAAAACTTA TCACATTCAT GACGCGTATA CAACGAAAGC TCGCTTGATT CCAGGGTATA 3346
EGD-e lmo0327  agcattacca acagttgatg caaaaactta tcacattcat gaggcttata caacgaaagc tcgcttgatt ccaggatata 3760
Consensus      AGCATTACCA ACAGTTGATG CAAAAACTTA TCACATTCAT GANGCNTATA CAACGAAAGC TCGCTTGATT CCAGGNTATA
Conservation

                                          3,780                                      3,800                                      3,820                                      3,840
N53-1          GCCTAGTAGC TGCACCAAAG AACCAAATAG GTGCTTTTGG AACAAGTGAT GTAACAGTCA ATTATGTGTA TAAAAAAGAT 3426
EGD-e lmo0327  gcctagtagc tgcaccaaag aaccaaatag gtgcttttgg aacaagtgat gtaacagtca attatgtgta taaaaaagat 3840
Consensus      GCCTAGTAGC TGCACCAAAG AACCAAATAG GTGCTTTTGG AACAAGTGAT GTAACAGTCA ATTATGTGTA TAAAAAAGAT
Conservation
```

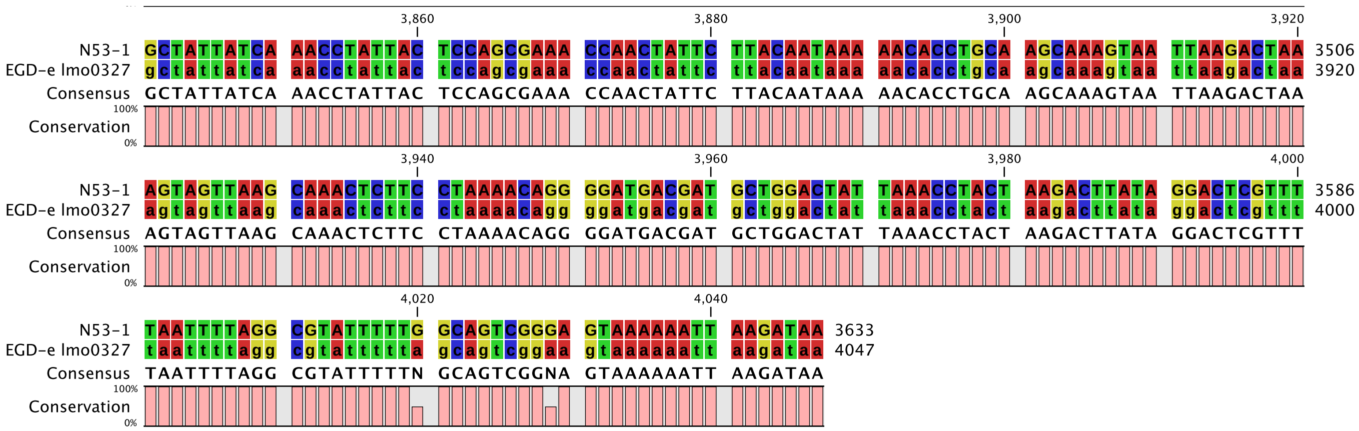

Figure S2. Nucleotide alignment of the *lmo0327* locus of strains N53-1 and EGD-e. The sequence for strain EGD-e was obtained from the ListiList database (http://genolist.pasteur.fr/ListiList/).

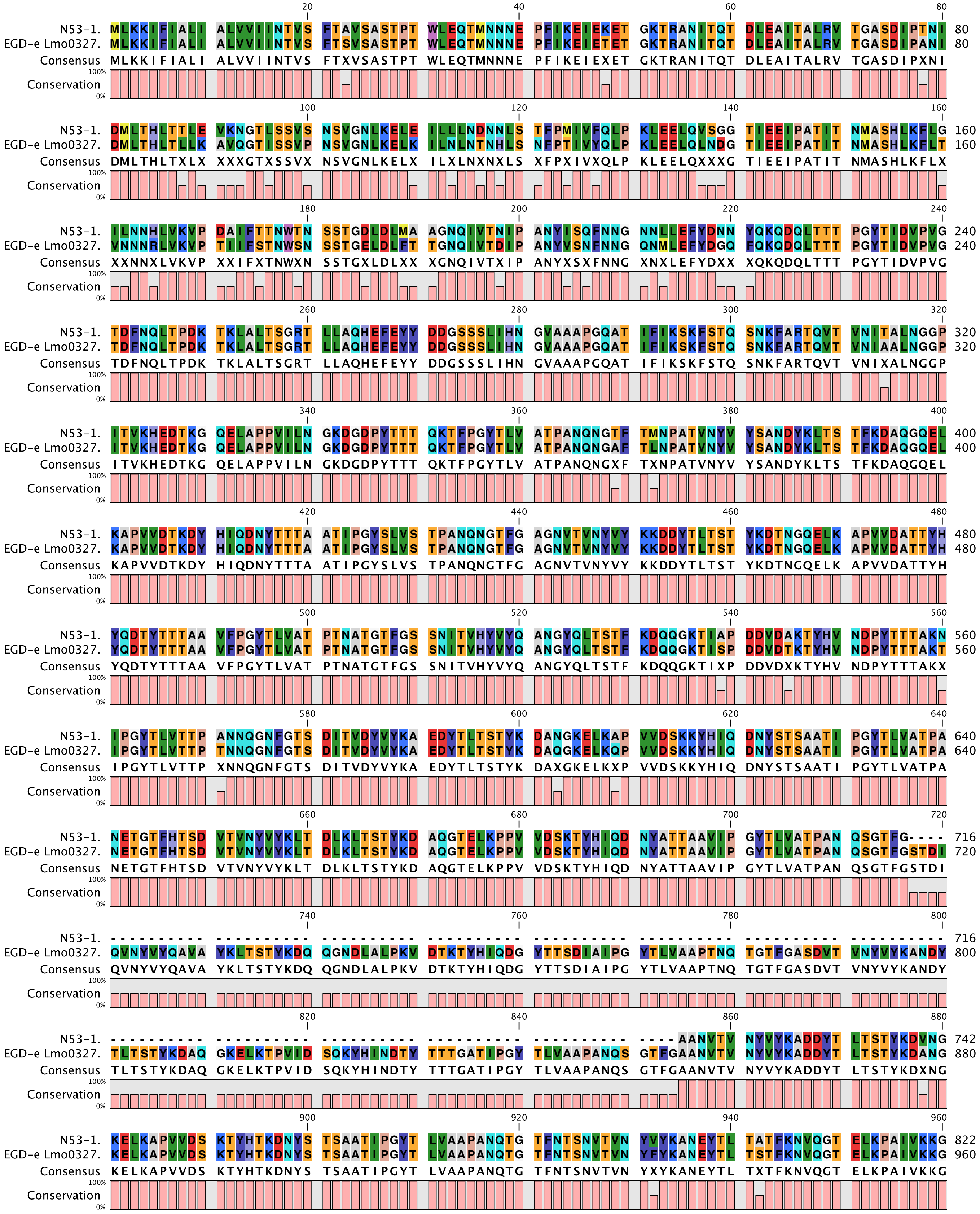
N53-1. MLKKIFIALI ALVVIINTVS FTAVSASTPT WLEQTMNNNE PFIKEIEKET GKTRANITQT DLEAITALRV TGASDIPTNI 80
EGD-e Lmo0327. MLKKIFIALI ALVVIINTVS FTSVSASTPT WLEQTMNNNE PFIKEIETET GKTRANITQT DLEAITALRV TGASDIPANI 80
Consensus MLKKIFIALI ALVVIINTVS FTXVSASTPT WLEQTMNNNE PFIKEIEXET GKTRANITQT DLEAITALRV TGASDIPXNI
N53-1. DMLTHLTTLE VKNGTLSSVS NSVGNLKELE ILLLNDNNLS TFPMIVFQLP KLEELQVSGG TIEEIPATIT NMASHLKFLG 160
EGD-e Lmo0327. DMLTHLTLLK AVQGTISSVP NSVGNLKELK ILNLNTNHLS NFPTIVYQLP KLEELQLNDG TIEEIPATIT NMASHLKFLT 160
Consensus DMLTHLTXLX XXXGTXSSVX NSVGNLKELX ILXLNXNXLS XFPXIVXQLP KLEELQXXXG TIEEIPATIT NMASHLKFLX
N53-1. ILNNHLVKVP DAIFTTNWTN SSTGDLDLMA AGNQIVTNIP ANYISQFNNG NNLLEFYDNN YQKQDQLTTT PGYTIDVPVG 240
EGD-e Lmo0327. VNNNRLVKVP TIIFSTNWSN SSTGELDLFT TGNQIVTDIP ANYVSNFNNG QNMLEFYDGQ FQKQDQLTTT PGYTIDVPVG 240
Consensus XXNNXLVKVP XXIFXTNWXN SSTGXLDLXX XGNQIVTXIP ANYXSXFNNG XNXLEFYDXX XQKQDQLTTT PGYTIDVPVG
N53-1. TDFNQLTPDK TKLALTSGRT LLAQHEFEYY DDGSSSLIHN GVAAAPGQAT IFIKSKFSTQ SNKFARTQVT VNITALNGGP 320
EGD-e Lmo0327. TDFNQLTPDK TKLALTSGRT LLAQHEFEYY DDGSSSLIHN GVAAAPGQAT IFIKSKFSTQ SNKFARTQVT VNIAALNGGP 320
Consensus TDFNQLTPDK TKLALTSGRT LLAQHEFEYY DDGSSSLIHN GVAAAPGQAT IFIKSKFSTQ SNKFARTQVT VNIXALNGGP
N53-1. ITVKHEDTKG QELAPPVILN GKDGDPYTTT QKTFPGYTLV ATPANQNGTF TMNPATVNYV YSANDYKLTS TFKDAQGQEL 400
EGD-e Lmo0327. ITVKHEDTKG QELAPPVILN GKDGDPYTTT QKTFPGYTLV ATPANQNGAF TLNPATVNYV YSANDYKLTS TFKDAQGQEL 400
Consensus ITVKHEDTKG QELAPPVILN GKDGDPYTTT QKTFPGYTLV ATPANQNGXF TXNPATVNYV YSANDYKLTS TFKDAQGQEL
N53-1. KAPVVDTKDY HIQDNYTTTA ATIPGYSLVS TPANQNGTFG AGNVTVNYVY KKDDYTLTST YKDTNGQELK APVVDATTYH 480
EGD-e Lmo0327. KAPVVDTKDY HIQDNYTTTA ATIPGYSLVS TPANQNGTFG AGNVTVNYVY KKDDYTLTST YKDTNGQELK APVVDATTYH 480
Consensus KAPVVDTKDY HIQDNYTTTA ATIPGYSLVS TPANQNGTFG AGNVTVNYVY KKDDYTLTST YKDTNGQELK APVVDATTYH
N53-1. YQDTYTTTAA VFPGYTLVAT PTNATGTFGS SNITVHYVYQ ANGYQLTSTF KDQQGKTIAP DDVDAKTYHV NDPYTTTAKN 560
EGD-e Lmo0327. YQDTYTTTAA VFPGYTLVAT PTNATGTFGS SNITVHYVYQ ANGYQLTSTF KDQQGKTISP DDVDTKTYHV NDPYTTTAKT 560
Consensus YQDTYTTTAA VFPGYTLVAT PTNATGTFGS SNITVHYVYQ ANGYQLTSTF KDQQGKTIXP DDVDXKTYHV NDPYTTTAKX
N53-1. IPGYTLVTTP ANNQGNFGTS DITVDYVYKA EDYTLTSTYK DANGKELKAP VVDSKKYHIQ DNYSTSAATI PGYTLVATPA 640
EGD-e Lmo0327. IPGYTLVTTP TNNQGNFGTS DITVDYVYKA EDYTLTSTYK DAQGKELKQP VVDSKKYHIQ DNYSTSAATI PGYTLVATPA 640
Consensus IPGYTLVTTP XNNQGNFGTS DITVDYVYKA EDYTLTSTYK DAXGKELKXP VVDSKKYHIQ DNYSTSAATI PGYTLVATPA
N53-1. NETGTFHTSD VTVNYVYKLT DLKLTSTYKD AQGTELKPPV VDSKTYHIQD NYATTAAVIP GYTLVATPAN QSGTFG---- 716
EGD-e Lmo0327. NETGTFHTSD VTVNYVYKLT DLKLTSTYKD AQGTELKPPV VDSKTYHIQD NYATTAAVIP GYTLVATPAN QSGTFGSTDI 720
Consensus NETGTFHTSD VTVNYVYKLT DLKLTSTYKD AQGTELKPPV VDSKTYHIQD NYATTAAVIP GYTLVATPAN QSGTFGSTDI
N53-1. ---------- ---------- ---------- ---------- ---------- ---------- ---------- ---------- 716
EGD-e Lmo0327. QVNYVYQAVA YKLTSTYKDQ QGNDLALPKV DTKTYHIQDG YTTSDIAIPG YTLVAAPTNQ TGTFGASDVT VNYVYKANDY 800
Consensus QVNYVYQAVA YKLTSTYKDQ QGNDLALPKV DTKTYHIQDG YTTSDIAIPG YTLVAAPTNQ TGTFGASDVT VNYVYKANDY
N53-1. ---------- ---------- ---------- ---------- ---------- ----AANVTV NYVYKADDYT LTSTYKDVNG 742
EGD-e Lmo0327. TLTSTYKDAQ GKELKTPVID SQKYHINDTY TTTGATIPGY TLVAAPANQS GTFGAANVTV NYVYKADDYT LTSTYKDANG 880
Consensus TLTSTYKDAQ GKELKTPVID SQKYHINDTY TTTGATIPGY TLVAAPANQS GTFGAANVTV NYVYKADDYT LTSTYKDXNG
N53-1. KELKAPVVDS KTYHTKDNYS TSAATIPGYT LVAAPANQTG TFNTSNVTVN YVYKANEYTL TATFKNVQGT ELKPAIVKKG 822
EGD-e Lmo0327. KELKAPVVDS KTYHTKDNYS TSAATIPGYT LVAAPANQTG TFNTSNVTVN YFYKANEYTL TSTFKNVQGT ELKPAIVKKG 960
Consensus KELKAPVVDS KTYHTKDNYS TSAATIPGYT LVAAPANQTG TFNTSNVTVN YXYKANEYTL TXTFKNVQGT ELKPAIVKKG
Conservation

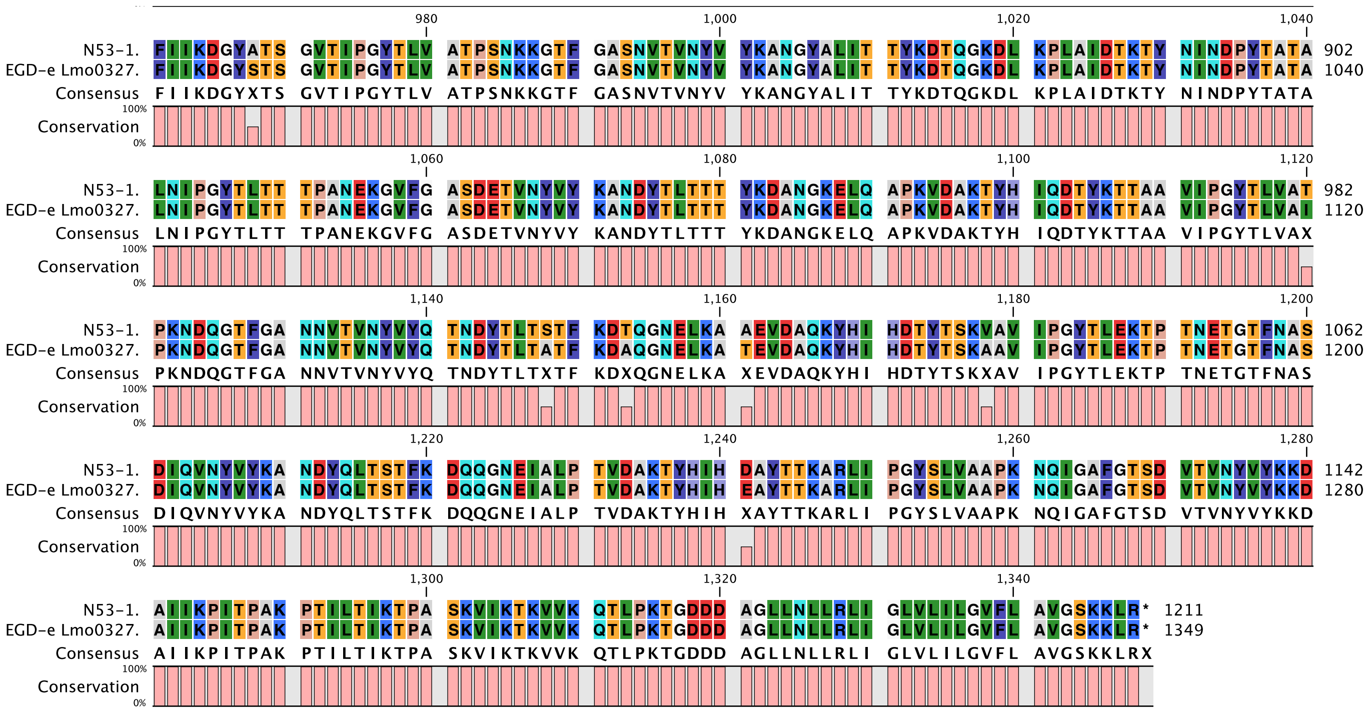

Figure S3. Protein alignment of the predicted sequences of Lmo0327 of strains N53-1 and EGD-e.
